# Integrated Multi-Omic Analyses Reveal Gender-Specific Molecular Mechanisms Regulating Growth and Muscle Development in Zhongshan Partridge Ducks

**DOI:** 10.3390/biom16091335

**Published:** 2026-09-14

**Authors:** Tong Ouyang, Xiaohuan Chao, Yingshan Yin, Congliang Ji, Xuerong Ma, Youcheng He, Tianxi Cui, Haoqi Zhong, Zhou Wu, Qingbin Luo

**Affiliations:** 1 College of Animal Science, South China Agricultural University, Guangzhou 510642, China; 13035785101@163.com (T.O.); xhchao@scau.edu.cn (X.C.); h18303943664@163.com (Y.H.); 13453623066@163.com (T.C.); haoqizhong@stu.scau.edu.cn (H.Z.); 2 State Key Laboratory of Livestock and Poultry Breeding, South China Agricultural University, Guangzhou 510642, China; 3 Zhongshan Agricultural Science and Technology Extension Center, Zhongshan 528400, China; jc_12323@163.com; 4 Wens Foodstuff Group Co., Ltd., Yunfu 527400, China; yzjcl@126.com (C.J.); ma15184834646@163.com (X.M.)

**Keywords:** Zhongshan Partridge duck, multi-omics, growth, pectoral muscle, GWAS, transcriptome, untargeted metabolome, sex dimorphism, growth curve

## Abstract

Zhongshan Partridge duck is a unique indigenous dual-purpose waterfowl native to China’s Pearl River Delta. This once endangered breed has been restored through conserved breeding. Its slow growth and low breast muscle yield limit industrial application, and obvious sexual dimorphism in pectoral muscle growth and metabolism lacks systematic molecular explanation. Herein, we combined GWAS, pectoral RNA-seq and untargeted metabolome to dissect regulatory networks of growth and carcass traits using 365 standardized ducks. Ten growth and carcass phenotypes were collected, and the Gompertz model fitted growth trajectories with mean R^2^ > 0.98. Critical trait-related genes, including *FTO*, *ACVR2B* and *IGFBP3*, were identified by GWAS. We detected 313 differentially expressed genes (DEGs) showed differentially expressed pattern between high- and low-breast-weight groups in males and 309 DEGs in females. Distinct sex-specific metabolic patterns were observed: males depended on pentose phosphate and glycine–serine–threonine pathways for muscle protein deposition, whereas females utilized fatty acid β-oxidation for energy and lipid control. Lipid gene *ECHS1* and growth factor *PRDM16* were identified as candidate regulators mediating sexually dimorphic breast muscle development. Joint enrichment of DEGs and differentially expressed metabolites (DEMs) pinpointed key pathways for myogenesis. This study elucidates multi-level sexual dimorphism in duck pectoral development, supplies molecular markers for indigenous duck conservation and breeding, and advances our understanding of poultry growth regulation.

## 1. Introduction

Meat duck production is a critical component of the global animal husbandry sector, and the meat duck industry serves as a pillar of the waterfowl farming industry, especially in China, with its industrial scale and output value rising year by year. In 2025, the slaughter volume of commercial meat ducks in China reached 4.382 billion, with a total industrial output value of 116.014 billion RMB [1]. The Zhongshan Partridge duck is a dual-purpose (egg and meat) indigenous breed existing over 600 years of low-intensity farming in the Pearl River Delta region, and it is the only native waterfowl breed named after the Zhongshan region, Guangdong Province. In the mid-to-late 20th century, affected by the large-scale promotion of fast-growing commercial meat-type ducks, the population of Zhongshan Partridge ducks declined significantly, and the breed was classified as an endangered indigenous breed [2]. Subsequently, the local research center and research institute carried out long-term multi-generational conservation and rejuvenation programs, and the breed was re-introduced in the Chinese National Catalog of Livestock and Poultry Genetic Resources Protection in 2024 [3,4]. This breed features prominent local germplasm characteristics such as crude feed tolerance, strong adaptability and resilience. However, compared with fast-growing commercial duck breeds, it typically exhibits a slower growth rate and lower pectoral muscle yield, which limits its industrial utilization and economic benefits [5,6]. Growth and carcass traits are core economic indicators for duck breeding. Body weight at different days of age and stage-specific weight gain determine production costs and turnover efficiency, while carcass weight and pectoral muscle weight are directly linked to industrial economic benefits [7,8]. Therefore, identifying key genes and metabolic pathways regulating growth and pectoral muscle development of Zhongshan Partridge ducks in this study can provide a theoretical foundation for the genetic improvement of these valuable local genetic resources.

In poultry research, genome-wide association study (GWAS) has become a classic approach for exploring genetic variations underlying complex quantitative traits at the DNA level, enabling efficient mapping of candidate genes associated with growth and carcass traits. To date, this approach has been applied in multiple duck breeds such as Pekin ducks and Sichuan Ma ducks, successfully mapping a set of functional candidate genes regulating growth and carcass traits [6,9,10], whereas RNA-seq enables high-throughput detection of gene expression profiles in specific tissues. Through differential expression analysis, key functional genes and pathways regulating trait development can be screened, establishing a functional link between genetic information and expression profiles, and has been widely used in studies to investigate the mechanism of poultry muscle development [11,12]. Furthermore, metabolomics has been extensively adopted in comparative studies of muscle traits among breeds with different growth performances. It systematically elucidates the metabolic characteristics of muscle development and provides direct phenotypic evidence for dissecting trait mechanisms [13,14,15,16]. Nevertheless, single-omics data can only dissect partial links of trait regulation from a single molecular perspective, making it difficult to fully elucidate the multi-dimensional regulatory cascade and global regulatory network of complex growth traits [17,18]. In addition, multi-omics integrated analysis has emerged as a mainstream strategy for deciphering the molecular mechanisms underlying complex traits in livestock and poultry, which can systematically construct a regulatory network across multiple interacting layers, including genetic variation, gene expression and metabolic regulation, substantially improving the efficiency and accuracy of candidate gene and functional pathways detection [19,20].

Previous studies showed that pronounced sexual dimorphism exists in the growth performance and pectoral muscle development of domestic ducks. Male ducks generally exhibit faster growth rates and superior hypertrophic muscle development, whereas female ducks tend to display steadier growth rhythms and higher intramuscular fat deposition, leading to marked sexual differences in final body weight, breast muscle weight, and meat quality [21,22]. RNA-seq and phenotypic analyses have preliminarily revealed sex-dependent differences in duck muscle fiber development and energy metabolism [23]. However, most available studies are limited to RNA-seq profiling or macroscopic phenotypic comparison. The integrated multi-omics regulatory mechanism underlying sexual dimorphism in duck breast muscle development, particularly the key genes and metabolic crosstalk driving sex-specific growth patterns in indigenous duck breeds, remains poorly understood. Therefore, expanding multi-omics evidence to decode the molecular basis of sex-specific breast muscle development is essential for advancing duck muscle biology and supporting precise genetic improvement of indigenous duck germplasm.

To expand the comprehensive understanding of growth and carcass traits of indigenous Ma ducks, this study used the core conservation population of Zhongshan Partridge ducks as the experimental cohort. We systematically measured core growth traits including body weight at hatch, 14, 28, 42 and 70 days of age, as well as carcass weight and pectoral muscle weight at 70 days of age. The measurement fully covers the entire growth cycle from hatching to the typical market age, overcoming the limitation of single-time-point phenotyping [9], and enabling simultaneous dissection of the genetic basis of both dynamic growth processes and carcass traits [6]. In total, 365 Zhongshan Partridge ducks were phenotyped and genotyped, and subsequently used for genome-wide association analysis, among which 28 representative individuals were further selected for transcriptomic and untargeted metabolomic profiling for multi-omics integration. Through cross-validation of multi-omics data, key pathways regulating pectoral muscle development and growth performance of Zhongshan Partridge ducks were highlighted. By multi-omics cross-validation, we aimed to identify key pathways and candidate genes responsible for pectoral muscle development and growth performance in Zhongshan Partridge ducks. Overall, this work intends to provide candidate molecular resources for functional-gene exploration and molecular-assisted breeding of this indigenous duck breed. Furthermore, a major objective of the present study is to clarify the multi-omics molecular basis underlying sex-specific differences in pectoral muscle development, so as to reveal the key genes and metabolic pathways driving sexual dimorphism in indigenous duck muscle growth.

## 2. Materials and Methods

### 2.1. Experimental Animals and Sample Collection

The core breeding population of Zhongshan Partridge ducks used in this study was sourced from the Zhongshan Partridge Duck Conservation Farm in Guangdong Province, China. Five consecutive batches of hatched Zhongshan Partridge ducks were selected as experimental subjects, with a hatching interval of 14 ± 1 day between adjacent batches. All hatching eggs were derived from the same breeder flock of a single grandparent breeder farm to ensure a consistent genetic background. A total of 365 healthy Zhongshan Partridge ducks (172 males and 193 females) were included in the experiment.

All ducks were reared under identical feeding and management conditions with water-based pen housing, with ad libitum access to feed and water throughout the trial. A phase-feeding strategy was adopted using commercial compound feeds. During the brooding period (1–28 days of age), ducklings were fed starter compound feed (metabolizable energy: 2850 kcal/kg, product No. 548); during the growing period (29–70 days of age), ducks were fed grower compound feed (metabolizable energy: 2900 kcal/kg, product No. 545). All feeds were purchased from Guangdong Guangda Bio-Nutrition Technology, Jiangmen, China. The main nutritional components are provided in Table S1.

Individual body weight was recorded on the day of hatching and at 14, 28, 42, and 70 days of age, respectively, in the morning under fasting conditions, using an electronic balance with a precision of 1 g. The recorded traits were defined as hatch weight (BW1), body weight at 14 days (BW14), body weight at 28 days (BW28), body weight at 42 days (BW42), and body weight at 70 days (BW70). Based on these measurements, absolute weight gain (AWG) for each growth stage was calculated: AWG from hatching to 28 days (AWG-28d), AWG from 28 to 42 days (AWG-28d to 42d), and AWG from 42 to 70 days (AWG-42d to 70d).

At 70 days of age, all ducks were fasted for 12 h (with free access to water) and slaughtered via carotid artery exsanguination. Carcass weight (CW) was determined immediately after slaughter. The bilateral pectoralis major and pectoralis minor muscles were completely dissected, and total breast muscle weight (BMW) was measured using an electronic balance with a precision of 0.01 g.

Before slaughter, 2 mL of venous blood was collected from all 365 ducks, treated with EDTA K2 as an anticoagulant, mixed by gentle inversion, and stored temporarily at −20 °C for subsequent DNA extraction and whole-genome resequencing.

From the 365 ducks, 28 individuals were selected to form high- and low-phenotype groups based on 70-day breast muscle weight. To minimize batch effects, selection was restricted to a single batch. Within this batch, male and female ducks were ranked separately by phenotypic value in descending order, and the top 7 and bottom 7 individuals from each sex were selected. This yielded four treatment groups: male high group (MH), male low group (ML), female high group (FH), and female low group (FL), with 7 biological replicates per group. This stringent phenotype-based sampling design was applied to improve statistical power for capturing molecular signatures associated with divergent pectoral muscle performance. The sample size ensured analytical power for transcriptomic and metabolomic screening of candidate genes and metabolites. Considering strong confounding effects derived from sex, only within-group comparisons were implemented, while cross-sex contrasts were not specifically investigated. The phenotype-driven signatures from male and female groups were compared in the result interpretation.

Within 15 min post-slaughter, approximately 2 g of tissue from the middle section of the pectoralis major muscle was collected from the 28 selected ducks. Residual blood was quickly rinsed off with pre-chilled sterile normal saline, and surface moisture was blotted dry with sterile filter paper. Each sample was divided into two 1 g aliquots and immediately snap-frozen in liquid nitrogen for subsequent RNA-seq and untargeted metabolomics profiling.

### 2.2. Whole-Genome Sequencing

Genomic DNA was extracted using a high-throughput magnetic bead method. DNA purity was verified by ultraviolet spectrophotometry, with an OD_260_/_280_ ratio in the range of 1.8–2.0 and an OD_260_/_230_ ratio > 1.8. DNA integrity was assessed via 1% agarose gel electrophoresis, with qualified samples required to show a clear main band without obvious degradation or trailing. DNA samples that passed quality control were used for subsequent sequencing library construction.

Qualified genomic DNA was fragmented into 300–500 bp fragments by ultrasonication. Paired-end sequencing libraries were constructed through sequential steps of end repair, 3′ A-tailing, sequencing adapter ligation, and PCR enrichment. After accurate quantification via qPCR and fragment distribution validation, the libraries were subjected to paired-end 150 bp whole-genome resequencing on the MGI DNBSEQ-T7 high-throughput sequencing platform, with a sequencing depth of approximately 10× per sample to ensure the accuracy of variant detection.

Quality control of raw sequencing reads was performed using Fastp software (v0.23.2). Reads containing adapter sequences, with an N-base ratio > 10%, or with a proportion of low-quality bases (quality value < 20) >50% were removed to obtain clean reads. Q20, Q30, and GC content were calculated to evaluate data quality. Clean reads were aligned to the duck reference genome GCF_003850225.1 (IASCAAS_PekingDuck_PBH1.5) using the BWA-MEM algorithm. Sorting and format conversion were completed via SAMtools. PCR duplicates were marked and removed using Picard tools. Base quality score recalibration (BQSR) was performed using GATK software (4.2.6.1) to obtain high-quality aligned BAM files.

Population variant calling was performed using the GATK HaplotypeCaller pipeline. GVCF files were generated for each sample, and joint genotyping was conducted to obtain the raw set of single nucleotide polymorphism (SNP) and insertion/deletion (InDel). Strict quality control of raw variants was performed using PLINK software (v1.9), with the following filtering criteria: variant call rate > 0.9 (missing rate < 0.1); minor allele frequency (MAF) > 0.05; Hardy–Weinberg equilibrium test *p* > 1 × 10^−6^. The final high-quality SNP dataset was used for subsequent genome-wide association analysis.

### 2.3. RNA-seq

Total RNA was extracted from breast muscle using the TRIzol method. For quality validation, RNA purity was determined by ultraviolet spectrophotometry, with an OD_260_/_280_ ratio of 1.8–2.1 and an OD_260_/_230_ ratio ≥ 1.8. RNA integrity was assessed using an Agilent 2100 Bioanalyzer, with an RNA integrity number (RIN) ≥ 7.0 and a 28S/18S ratio ≥ 1.0. Agarose gel electrophoresis was performed to verify band clarity and confirm the absence of degradation and genomic DNA contamination.

Total RNA was then used for mRNA enrichment via Oligo(dT) magnetic beads, followed by fragmentation into 200–300 bp short fragments using fragmentation buffer. Double-stranded cDNA was synthesized using the short mRNA fragments as templates. cDNA sequencing libraries were constructed through end repair, A-tailing, sequencing adapter ligation, and PCR amplification. After passing qPCR quantification and fragment distribution validation, the libraries were subjected to paired-end 150 bp high-throughput sequencing on the Illumina NovaSeq 6000 platform to obtain raw sequencing reads.

Quality control of raw sequencing data was performed using Fastp software. Reads containing sequencing adapters, with an N-base ratio > 5%, or with a proportion of low-quality bases (quality value < 20) > 50% were removed to generate clean reads. Q20, Q30, and GC content were calculated to evaluate data quality. Clean reads were aligned to the duck reference genome GCF_003850225.1 (IASCAAS_PekingDuck_PBH1.5) using HISAT2 software (v2.2.1). Alignments were sorted and format-converted via SAMtools for subsequent gene expression quantification.

Based on the gene annotation file (GFF format) corresponding to the reference genome, featureCounts software (v2.0.3) was used to quantify reads mapped to the exon region of each gene. A raw read count matrix for each sample was obtained for subsequent statistical analysis.

### 2.4. Untargeted Metabolomics Profiling

Pooled quality control (QC) samples were prepared to evaluate the stability of the detection system. An appropriate amount of frozen breast muscle tissue was weighed, mixed with a pre-chilled methanol–acetonitrile–water solution (volume ratio 2:2:1), and homogenized by grinding at low temperature. After vortex mixing, ultrasonic extraction was performed on ice for 20 min, followed by protein precipitation by standing at −20 °C. After centrifugation at 12,000 r/min for 15 min at 4 °C, the supernatant was collected and vacuum freeze-dried. Before injection, samples were reconstituted with a methanol–water solution, vortexed, and centrifuged again; the supernatant was collected for on-machine detection.

Untargeted metabolomics detection was performed on an ultra-high-performance liquid chromatography coupled with quadrupole-electrostatic field orbitrap mass spectrometry (UHPLC-Q-Exactive Orbitrap MS) platform, and data were acquired in both positive and negative ion modes. For chromatographic conditions, a C18 reversed-phase chromatographic column was used, with a column temperature of 40 °C, a flow rate of 0.3 mL/min, and an injection volume of 2 μL; an aqueous–organic gradient elution program was set. For mass spectrometry conditions, a full MS scan combined with data-dependent MS/MS scan mode was adopted, with a mass-to-charge ratio scanning range of *m*/*z* 100–1000; corresponding spray voltage, sheath gas, and auxiliary gas parameters were set.

Raw mass spectrometry data were processed using Progenesis QI software (v2.4) for peak alignment, peak extraction, denoising, and peak area quantification. Metabolite characteristic peaks with a detection rate > 80% in QC samples were retained, and unstable signals with large system fluctuations were removed. Metabolite structural identification was completed by matching primary exact mass values and secondary fragmentation spectra with the HMDB, KEGG, and local metabolite databases. Finally, a relative quantification matrix of metabolites was obtained for subsequent statistical analysis.

### 2.5. Statistical Analysis

#### 2.5.1. Genome-Wide Association Analysis

As for GWAS analysis, to mitigate confounding between genetic relatedness and causal variants, genome-wide association analysis was performed using the FarmCPU (Fixed And Random Model Circuitous Probability Unification) model implemented in the R package Rmvp (v1.0.6) [24,25]. Unlike the traditional Mixed Linear Model (MLM), which constructs a single variance component using genome-wide markers, FarmCPU iterates between a Fixed Effect Model (FEM) and a Random Effect Model (REM).

The FEM step conducts genome-wide SNP scanning, where pseudo-QTNs identified from the preceding REM step are incorporated as fixed covariates to absorb known genetic signals. This strategy reduces polygenic background noise and improves statistical power for detecting new trait-associated SNPs. In the FEM step, phenotypes are regressed on candidate SNPs and previously selected pseudo-quantitative trait nucleotides (pseudo-QTNs), with the latter serving as fixed covariates to explain phenotypic variation: y = Xβ + Sµ + Qα + e, where y is the vector of phenotypic values; X is the design matrix for fixed effects; β is the vector of fixed effect coefficients; S is the genotype vector or matrix of the SNP under test; μ is the vector of random additive genetic effects; Q is the matrix of pseudo-QTNs; α is the vector of effects for pseudo-QTNs; and e is the vector of residual errors.

In the REM step, only pseudo-QTNs are used to construct a compressed kinship matrix (K_c_) to estimate the polygenic background: Y = Xβ + Zµ + e, µ~N(0, K_c_σ^2^_g_), where Z is the incidence matrix linking individuals to random effects; K_c_ is the compressed kinship matrix constructed based on pseudo-QTNs; and σ^2^_g_ is the genetic variance component.

This iterative process continues until convergence, effectively separating the effects of confounding loci from the polygenic background. Population structure, kinship matrix, batch effect, and sex effect were included as covariates in the GWAS model. The significance threshold was set at *p* < 1 × 10^−5^. Manhattan plots and quantile–quantile (QQ) plots were generated using the R package ggplot2. Notably, *p* < 1 × 10^−5^ was predefined as the suggestive genome-wide threshold in our analysis, which is commonly used for duck GWAS analyses for growth-related traits.

#### 2.5.2. Differentially Expressed Gene Detection

Differential expression analysis of the expression count matrix was performed using the R package DESeq2. This method is based on a generalized linear model with a negative binomial distribution, and performs library size normalization and dispersion estimation for read counts. Fold change in gene expression between the high and low breast muscle groups was calculated, and significance *p*-values were obtained via the Wald test. Adjusted *p*-values (padj) were generated using the Benjamini–Hochberg (BH) method for multiple-testing correction. Significantly differentially expressed genes (DEGs) were defined as genes with padj < 0.05 and fold-change (FC) ≥ 1.2, corresponding to |log_2_FC| ≥log_2_ (1.2). KEGG pathway enrichment analysis was performed on the DEGs. The significance of enrichment between gene sets and functional terms was calculated using Fisher’s exact test, followed by BH correction for multiple testing. Functional terms and signaling pathways related to breast muscle development and muscle growth were screened with a threshold of adjusted *p* < 0.05. Enrichment analysis was implemented using the R package clusterProfiler.

Intersection analysis was performed between DEGs and GWAS candidate genes, visualized by a Venn diagram. The significance of the intersecting genes was verified using a hypergeometric distribution test to identify key genes associated with breast muscle traits at both the genomic variation and gene expression levels, which provide targets for multi-omics integrated analysis.

Protein–protein interaction (PPI) networks for DEGs from male and female groups were constructed separately using the STRING database (https://string-db.org/, accessed on 24 June 2026.) with Anas platyrhynchos as the reference species. Only interactions with a confidence score ≥ 0.7 (high-confidence interactions) were retained. Some DEGs lacked available protein-interaction evidence and were excluded from the network. The resulting networks were imported into Cytoscape software (v3.10.4) for visualization and topological parameter calculation. All gene nodes within each PPI network were retained for downstream analysis; degree values were calculated for every node to evaluate gene connectivity, and no additional filtering was performed to extract a subset of hub-gene candidates.

#### 2.5.3. Metabolomics Analysis

The relative quantification matrix of metabolites was preprocessed by Pareto scaling to eliminate the influence of magnitude differences among metabolites. Principal component analysis (PCA) was applied to observe the overall sample distribution, inter-group separation trends, and outlier samples. Orthogonal partial least squares discriminant analysis (OPLS-DA) was performed to maximize metabolic differences between groups. Model reliability was evaluated via 7-fold cross-validation and 200 permutation tests to avoid overfitting. The variable importance in projection (VIP) value was extracted to measure the contribution of each metabolite to inter-group differences. Differential metabolites were screened jointly by combining multivariate and univariate statistical results, with the following criteria: VIP ≥ 1 from the OPLS-DA model, *p* < 0.05 from an independent-samples *t*-test, and fold change (FC) ≥ 1.2 or FC ≤ 1/1.2. In this study, differential metabolites were screened using the criteria of VIP ≥ 1 from OPLS-DA and *p* < 0.05 from Student’s *t*-test. This filtering strategy has been widely adopted in untargeted metabolomic studies of livestock muscle traits. Nevertheless, we acknowledge that potential false-positive effects resulting from the absence of multiple-testing adjustment should be noted.

For both transcriptomic and metabolomic analyses, we performed within-sex comparisons only, MH-versus-ML and FH-versus-FL. These within-sex contrasts allow us to investigate molecular changes associated with breast muscle weight differences while keeping sex constant.

#### 2.5.4. Screening of Core Candidate Genes

Core candidate target genes were screened from the non-redundant candidate gene set derived from GWAS. Two-dimensional evidence was applied for screening: (1) genes located within or near significant GWAS signal regions associated with breast-muscle-related traits; (2) genes that have previously been reported in the published literature to participate in skeletal muscle development, proliferation or differentiation. Genes satisfying both criteria were recognized as core candidate targets for subsequent interpretation.

#### 2.5.5. Software and Database Resources

Transcriptomic and untargeted metabolomic statistical analyses were performed using online OmicStudio tools (Lianchuan Biotechnology, Hangzhou, China; https://www.omicstudio.cn/tool, accessed on 11 July 2026). All additional statistical computations were implemented in R software (v4.2.1; https://www.r-project.org/, accessed on 9 June 2026). The GWAS was carried out using the rMVP R package (https://cran.r-project.org/web/packages/rMVP/, accessed on 9 June 2026). The DESeq2 R package (v1.38.3; https://bioconductor.org/packages/DESeq2/, accessed on 9 June 2026) was used for differential gene expression analysis. The protein–protein interaction network was constructed via the online STRING database (https://string-db.org/, accessed on 24 June 2026) and visualized in Cytoscape software (v3.10.4; https://cytoscape.org/, accessed on 24 June 2026). Functional enrichment analysis was based on the KEGG database (https://www.genome.jp/kegg/, accessed on 9 June 2026). Duck reference genome and gene annotation files were downloaded from the NCBI Assembly database (accession: GCF_003850225.1). Metabolite annotation was conducted using the HMDB database (https://hmdb.ca/, accessed on 9 June 2026).

### 2.6. Ethical Statement

All animal experiments were conducted in accordance with the Guide for the Care and Use of Laboratory Animals and approved by the Laboratory Animal Ethics Committee organized by the Department of Science and Technology of Guangdong Province (approval No. SYXK-2024-0136, 31 March 2024).

## 3. Results

### 3.1. Descriptive Statistics of Phenotypic Data

To characterize the growth performance of Zhongshan Partridge ducks, seven growth and carcass traits were measured in this study, including hatch weight (BW1), body weight at 14 days of age (BW14), body weight at 28 days of age (BW28), body weight at 42 days of age (BW42), body weight at 70 days of age (BW70), carcass weight (CW), and breast muscle weight (BMW). Three stage-specific absolute weight gain traits were further calculated: absolute weight gain from hatching to 28 days of age (AWG-28d), absolute weight gain from 28 to 42 days of age (AWG-28d to 42d), and absolute weight gain from 42 to 70 days of age (AWG-42d to 70d).

Given the significant effect of sex on growth traits in waterfowl, descriptive statistics were performed independently for male and female populations (Table 1). The results showed that the mean hatch weight (BW1) was similar between male and female ducks. With increasing age, the difference in body weight between the two groups gradually emerged; the body weight of male ducks at 70 days of age reached 2322.00 g, which was slightly higher than that of female ducks (2214.54 g). In terms of stage weight gain, there was negligible difference in absolute weight gain between male and female ducks during the 28–42 day period. During the 42–70 day period, the mean absolute weight gain of male ducks was 708.00 g, which was significantly higher than that of female ducks (595.86 g). For carcass traits, the mean values of breast muscle weight and carcass weight in male ducks were both higher than those in female ducks, indicating that male ducks exhibit a superior growth rate and breast muscle deposition capacity in the late growth stage.

**Figure 1:**
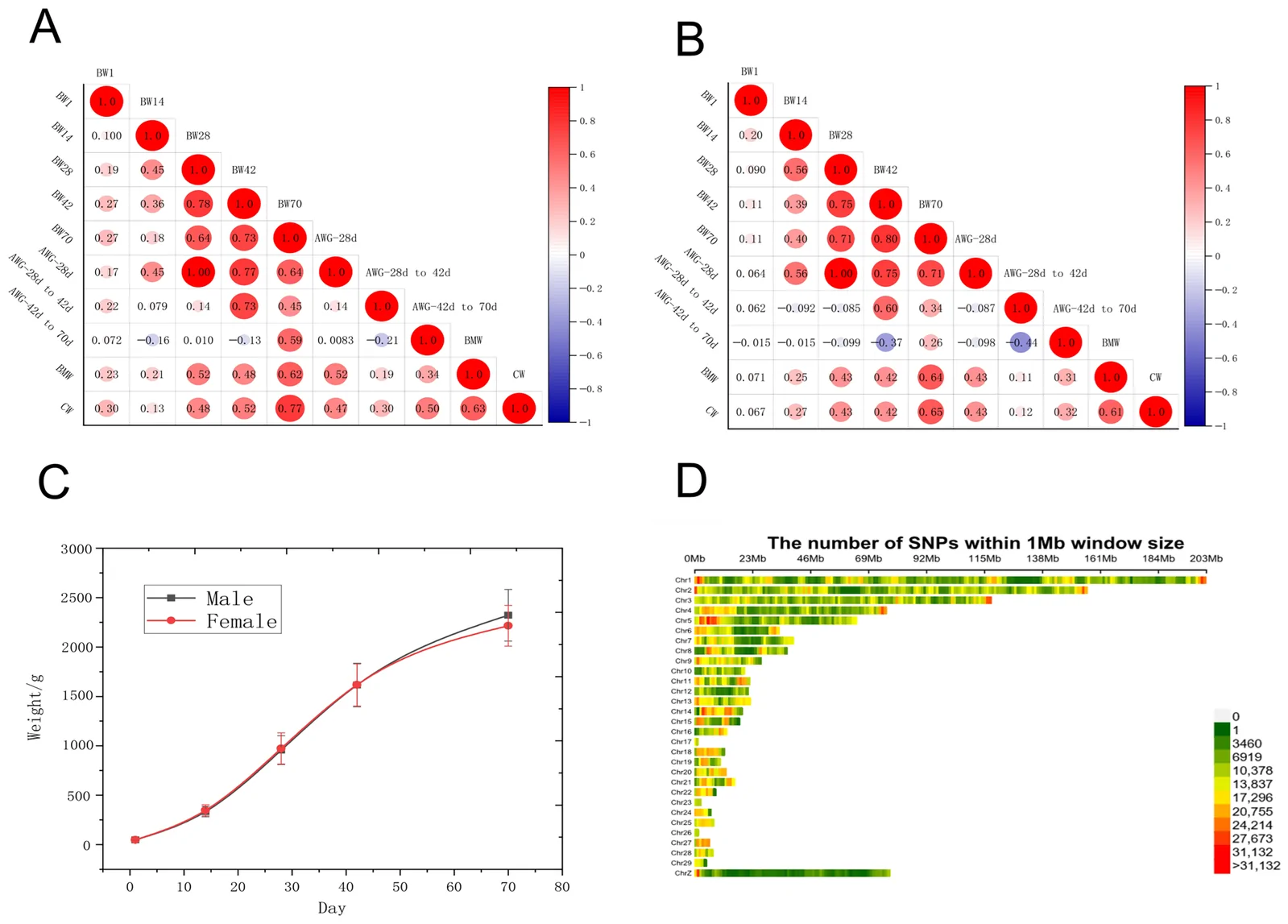
Phenotypic trait correlation, growth curve fitting, and genome-wide SNP density distribution of Zhongshan Partridge ducks. (**A**,**B**) Pearson correlation heatmaps of traits in the (**A**) male and (**B**) female duck populations. The color scale represents the Pearson correlation coefficient: darker red indicates a stronger positive correlation between traits, while darker blue indicates a stronger negative correlation. BW1, BW14, BW28, BW42, and BW70 represent body weight at hatch, 14, 28, 42, and 70 days of age, respectively; AWG represents average daily gain; BMW represents breast muscle weight; CW represents carcass weight. (**C**) Growth curve fitting of body weight from 1 to 70 days of age in Zhongshan Partridge ducks using the Gompertz model. The *x*-axis indicates age in days (d), and the *y*-axis indicates individual body weight in grams (g). Curves represent the average population growth trend, with error bars showing the standard deviation of body weight at each age. The red curve corresponds to male ducks and the black curve to female ducks. (**D**) Genome-wide SNP density distribution of Zhongshan Partridge ducks. The *x*-axis shows chromosomal physical position (Mb), and the *y*-axis shows chromosome numbers. The color gradient reflects the number of SNPs within a 1 Mb sliding window. The color bar on the right marks the corresponding SNP count ranges, with dark red colors indicating higher SNP density in the region.

To explore the relationship among growth and carcass traits, phenotypic correlation heatmaps were generated for male and female duck populations separately (Figure 1A,B). A larger Pearson correlation coefficient (r) indicates a stronger positive association between traits. In male ducks, generally positive correlations were observed across all measured traits (Figure 1A). Strong positive correlations were detected between BW28 and BW42 (r = 0.78), as well as between BW28 and BW70 (r = 0.64). BW42 exhibited strong positive correlations with BW70 (r = 0.73), AWG-28d (r = 0.77), and AWG-28d to 42d (r = 0.73). BW70 showed strong positive correlations with AWG-28d (r = 0.64), BMW (r = 0.62), and CW (r = 0.77). In female ducks (Figure 1B), BW28 was strongly positively correlated with BW42 (r = 0.75) and BW70 (r = 0.71). BW42 displayed strong positive correlations with BW70 (r = 0.80), AWG-28d (r = 0.75), and AWG-28d to 42d (r = 0.60). BW70 was strongly positively correlated with AWG-28d (r = 0.71), BMW (r = 0.64), and CW (r = 0.65). Overall, significant positive associations existed among body weight at different stages, stage-specific weight gain, breast muscle weight, and carcass weight.

To accurately characterize the growth and development patterns of male and female Zhongshan Partridge ducks, growth curves were constructed based on body weight data at 1, 14, 28, 42, and 70 days of age (Figure 1C). Three nonlinear growth models, namely the Logistic, Gompertz, and von Bertalanffy models, were fitted using Origin software (accessed on 10 June 2026). The fitting performance, growth inflection point, and asymptotic weight parameters of each model were compared (Table 2).

The growth trends of male and female ducks were generally consistent; the growth rate increased moderately from 1 to 28 days of age, and entered a rapid weight gain phase from 28 to 70 days of age. For male ducks, the adjusted coefficients of determination (adjusted R^2^) of all three nonlinear models were greater than 0.99. The Logistic model yielded an asymptotic body weight of 3125.50 g and an inflection point age of 42.02 days. The Gompertz model gave an asymptotic body weight of 2691.81 g and an inflection point age of 28.94 days. The von Bertalanffy model showed an asymptotic body weight of 3568.45 g and an inflection point age of 31.81 days. For female ducks, the adjusted R^2^ values of all three models also exceeded 0.99. The Logistic model produced an asymptotic body weight of 2822.71 g and an inflection point age of 38.06 days. The Gompertz model resulted in an asymptotic body weight of 2503.71 g and an inflection point age of 27.07 days. The von Bertalanffy model generated an asymptotic body weight of 3128.98 g and an inflection point age of 28.29 days.

Based on the comprehensive evaluation of adjusted R^2^ and residual sum of squares, the Gompertz model had an adjusted R^2^ closest to one and presented the optimal goodness of fit. Therefore, the Gompertz model is most suitable for describing the growth and development patterns of Zhongshan Partridge ducks.

### 3.2. Results of Genome-Wide Association Study (GWAS)

A total of 365 blood samples of Zhongshan Partridge ducks were collected via wing vein puncture. After quality control, sequence alignment, SNP calling, and strict filtering following standardized quality control criteria, 10,861,863 high-quality SNP markers were finally obtained. A genome-wide SNP density map was generated to visualize the distribution pattern of markers across chromosomes (Figure 1D). SNPs were unevenly distributed both among autosomes and within individual chromosomes. Large autosomes (chromosomes 1 to 3) had significantly higher overall SNP density than the remaining medium and small chromosomes, with extensive continuous highly polymorphic segments. Chromosomes 4 to 29 harbored medium-density SNPs only in local intervals, with sparse markers in most regions. The Z chromosome showed markedly lower overall SNP density than autosomes. In avian species, males carry the ZZ chromosomes and females carry the ZW chromosomes. The W chromosome is characterized by severe sequence degeneration, enrichment of repetitive sequences, and incomplete reference genome assembly, which makes it difficult to obtain reliably genotyped SNP markers from resequencing data. Accordingly, only the Z chromosome is presented in the SNP density map, and no valid SNPs were presented for the W chromosome. Subsequent GWAS was performed based on these SNP markers.

**Figure 2:**
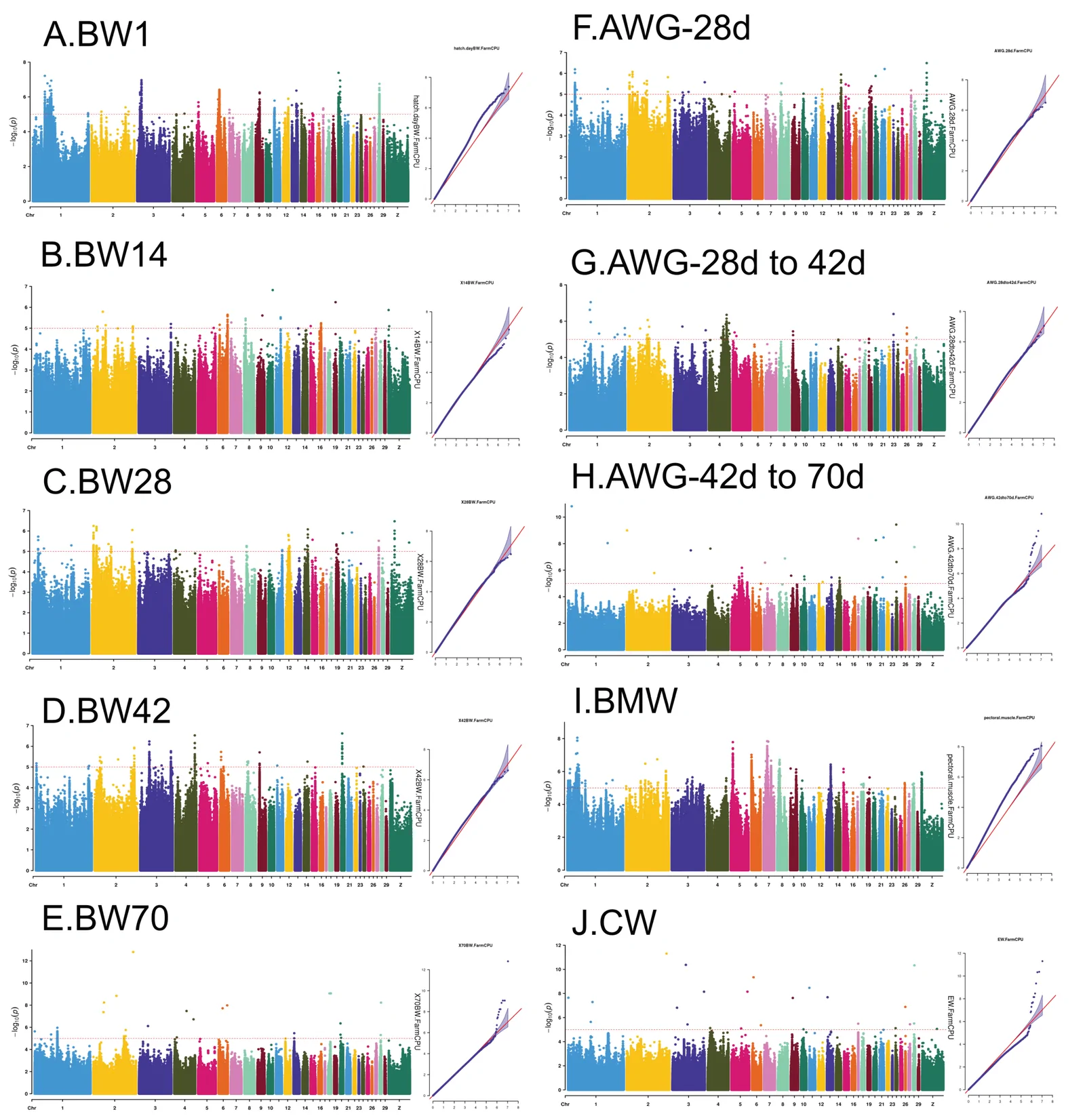
Manhattan plots of genome-wide association study (GWAS) for growth and carcass-related traits in Zhongshan Partridge ducks. (**A**–**J**) correspond to BW1, BW14, BW28, BW42, BW70, AWG-28d, AWG-28d to 42d, AWG-42d to 70d, BMW, and CW, respectively. Each dot represents a single SNP marker. The horizontal dashed line indicates the suggestive significance threshold (*p* < 1 × 10^−5^). In each panel, the *x*-axis of the Manhattan plot indicates chromosomal physical position (Mb), and the *y*-axis represents −log_10_ (*p*-value). Different colors along the x-axis represent different duck chromosomes. Significantly associated SNP loci are marked with colored scatter points. The small inset within each panel shows the corresponding Q-Q plot.

In the studied Zhongshan Partridge duck population, GWAS analyses identified 869 SNPs significantly associated with BW1, corresponding to 116 candidate genes (Figure 2A). For BW14, 45 significantly associated SNPs and 25 candidate genes were detected (Figure 2B). For BW28, 107 significantly associated SNPs and 37 candidate genes were identified (Figure 2C). For BW42, 131 significantly associated SNPs and 30 candidate genes were detected (Figure 2D). For BW70, 56 significantly associated SNPs and 23 candidate genes were found (Figure 2E). For AWG-28d, 108 significantly associated SNPs and 42 candidate genes were identified (Figure 2F). For AWG-28d to 42d, 122 significantly associated SNPs and 38 candidate genes were detected (Figure 2G). For AWG-42d to 70d, 46 significantly associated SNPs and 40 candidate genes were found (Figure 2H). For BMW, 1457 significantly associated SNPs and 146 candidate genes were identified (Figure 2I). For CW, 24 significantly associated SNPs and 31 candidate genes were detected (Figure 2J). The Q-Q plots for all ten phenotypic traits are presented in the insets of Figure 2A–J.

Notably, the number of significant SNPs detected for BMW was greater than that for other traits, which may suggest that breast muscle weight is regulated by a more abundant number of genomic loci. The complete list of significant SNPs is provided in Table S2. Among all annotated candidate genes, three functionally relevant genes were selected as core targets for further investigation, including *FTO*, which was identified within the significant interval for BW1, *ACVR2B* for AWG-28d to 42d, and *IGFBP3* for BMW.

**Figure 3:**
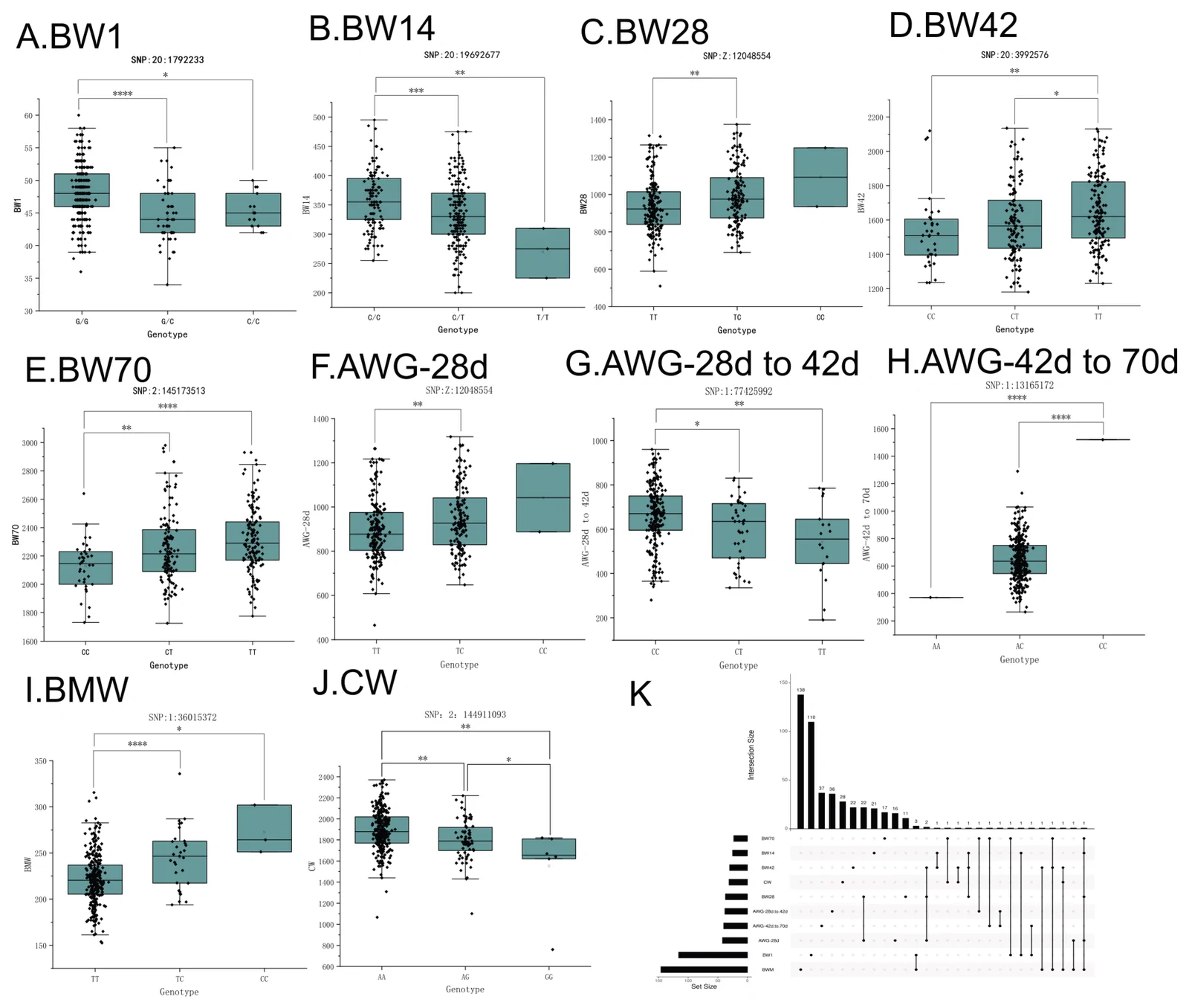
Box plots of associations between genotypes of significant SNP loci and growth and carcass traits. (**A**–**J**) correspond to BW1, BW14, BW28, BW42, BW70, AWG-28d, AWG-28d to 42d, AWG-42d to 70d, BMW, and CW, respectively. In each panel, the *x*-axis indicates SNP genotypes, and the *y*-axis indicates the phenotypic value of the trait. Box plots illustrate the differences in phenotypic distribution across different genotypes. (**K**) UpSet plot of intersecting GWAS candidate genes for 10 growth and carcass traits in Zhongshan Partridge ducks. The horizontal bars in the bottom-left panel represent the total number of candidate genes for each trait. The height of the vertical bars in the top panel indicates the number of shared genes corresponding to each trait combination. The connected black dots in the central matrix denote the set of traits included in each intersection. Asterisks denote significance levels: * *p* < 0.05, ** *p* < 0.01, *** *p* < 0.001, **** *p* < 0.0001.

Based on the significant SNPs identified by GWAS, we further investigated the associations between specific genotypes and phenotypic values, and screened functional markers with potential breeding application value. Locus 20:1792233 (G/C) was significantly associated with BW1 (*p* = 1.08 × 10^−7^) (Figure 3A). Individuals with the GG genotype had higher BW1, while those with the CC genotype had lower BW1 (Table S3). Locus 20:19692677 (C/T) was significantly associated with BW14 (*p* = 1.06 × 10^−5^) (Figure 3B). Individuals carrying the CC genotype exhibited higher BW14, whereas those with the TT genotype showed lower BW14. Locus Z:12048554 (T/C) was significantly associated with BW28 (*p* = 0.0016) (Figure 3C). The CC genotype corresponded to higher BW28, and the TT genotype corresponded to lower BW28. Locus 20:3992576 (C/T) was significantly associated with BW42 (*p* = 0.0028) (Figure 3D). Individuals with the TT genotype had higher BW42, while those with the CC genotype had lower BW42. Locus 2:145173513 (C/T) was significantly associated with BW70 (*p* = 8.08 × 10^−5^) (Figure 3E). The TT genotype was associated with higher BW70, and the CC genotype was associated with lower BW70. Locus Z:12048554 (T/C) was significantly associated with AWG-28d (*p* = 0.0027) (Figure 3F). Individuals with the CC genotype showed higher AWG-28d, whereas those with the TT genotype showed lower AWG-28d. Locus 1:77425992 (C/T) was significantly associated with AWG-28d to 42d (*p* = 0.0002) (Figure 3G). The CC genotype yielded higher AWG-28d to 42d, and the TT genotype yielded lower. Locus 1:13165172 (A/C) was significantly associated with AWG-42d to 70d (*p* = 1.65 × 10^−7^) (Figure 3H). Individuals carrying the CC genotype had higher AWG-42d to 70d, while those with the AA genotype had lower. Locus 2:144911093 (A/G) was significantly associated with CW (*p* = 1.41 × 10^−5^) (Figure 3I). The AA genotype corresponded to the higher CW, and the GG genotype corresponded to the lower CW. Locus 1:36015372 (T/C) was significantly associated with BMW (*p* = 1.46 × 10^−6^) (Figure 3J). Individuals with the CC genotype had higher BMW, whereas those with the TT genotype had lower BMW. Complete statistical data for all loci, including genotype sample size, phenotypic mean, and significance of analysis of variance (ANOVA), are provided in detail in Table S3.

Based on the significant SNPs identified by GWAS analysis, gene annotation was conducted for the 5 kb flanking regions upstream and downstream of loci, and the candidate genes for each trait were detected (Table S4). Results showed that a total of 116 candidate genes were annotated for BW1, among which *HABP2* exhibited the highest SNP enrichment in its genomic interval, harboring 54 significantly associated loci. For BW14, 25 candidate genes were annotated, with *VPS26A* showing higher SNP enrichment and carrying 15 significant loci. For BW28, 37 candidate genes were annotated, and *LOC101799054* was the gene with higher SNP enrichment, containing 27 significant loci. For BW42, 30 candidate genes were annotated, with *AARS2* presenting the highest SNP enrichment and harboring 30 significant loci. For BW70, 23 candidate genes were annotated, and *CCDC178* had the highest SNP enrichment with five significant loci.

For AWG-28d, 42 candidate genes were annotated, with *LOC101799054* showing the highest SNP enrichment and harboring 10 significant loci. For AWG-28d to 42d, 38 candidate genes were annotated, and *PPP2R2C* exhibited higher SNP enrichment, harboring 39 significant loci. For AWG-42d to 70d, 40 candidate genes were annotated, with *LOC101793679* showing the highest SNP enrichment and containing five significant loci. For CW, 31 candidate genes were annotated, and each gene carried only one significantly associated locus. For BMW, 146 candidate genes were annotated, and *LOC101794158* displayed higher SNP enrichment in its interval, with 109 significant loci.

An UpSet plot was used to visualize the intersection distribution pattern of GWAS-annotated candidate genes for the 10 growth and carcass traits of Zhongshan Partridge ducks (Figure 3K). There were 22 intersecting genes between BW28 and AWG-28d. Three overlapping genes were identified between BW1 and BMW, namely *LOC101798288*, *HS6ST3*, and *LOC101797392*. Two intersecting genes, *HOMER3* and *LRRC7*, were shared by BW28, BW42, and AWG-28d. The overlapping gene between BW1 and BW14 was *HIBADH*; that between BW1 and BW70 was *SBN02*; and that between BW1 and AWG-42d to 70d was *LOC101799201*. *ZHX2* was the common intersecting gene among BW14, BW28, and BW42. *NSMCE2* was the overlapping gene shared by BW14, BW28, BW70, AWG-28d, and BMW. *ACADSB* was the intersecting gene between BW14 and BW42.

*FGFRL1* was the common gene among BW42, BW70, and BMW. *MTSS1* was the overlapping gene among BW42, CW, and BMW. The intersecting gene between BW42 and CW was *HETL*, and that between BW42 and BMW was *DAB1*. The overlapping gene between BW70 and AWG-28d to 42d was *ARHGAP24*; that between BW70 and AWG-42d to 70d was *GRID2*; and that between BW70 and CW was *NYX*. *FAM124A* was the intersecting gene between AWG-28d and BMW. *ATP12A1* was the common overlapping gene between AWG-28d to 42d and AWG-42d to 70d.

Based on the predefined two-dimensional screening criteria described in Section 2, *FTO*, *ACVR2B* and *IGFBP3* were obtained as core candidate genes. Specifically, *FTO* was associated with hatch weight, *ACVR2B* was associated with absolute weight gain from 28 to 42 d, and *IGFBP3* was associated with breast muscle weight.

### 3.3. RNA-Seq Data Analysis

To investigate the gene expression basis of breast muscle phenotypic differentiation in Zhongshan Partridge ducks, RNA-seq was performed on 28 breast muscle samples. A total of 1,129,760,022 raw reads were generated. After filtering out low-quality sequences and removing adapter-contaminated sequences, 1,085,099,050 clean reads were obtained. The Q20 and Q30 values of all samples exceeded 95%, indicating reliable sequencing quality. The complete quality control data for all 28 samples are provided in Table S5.

**Figure 4:**
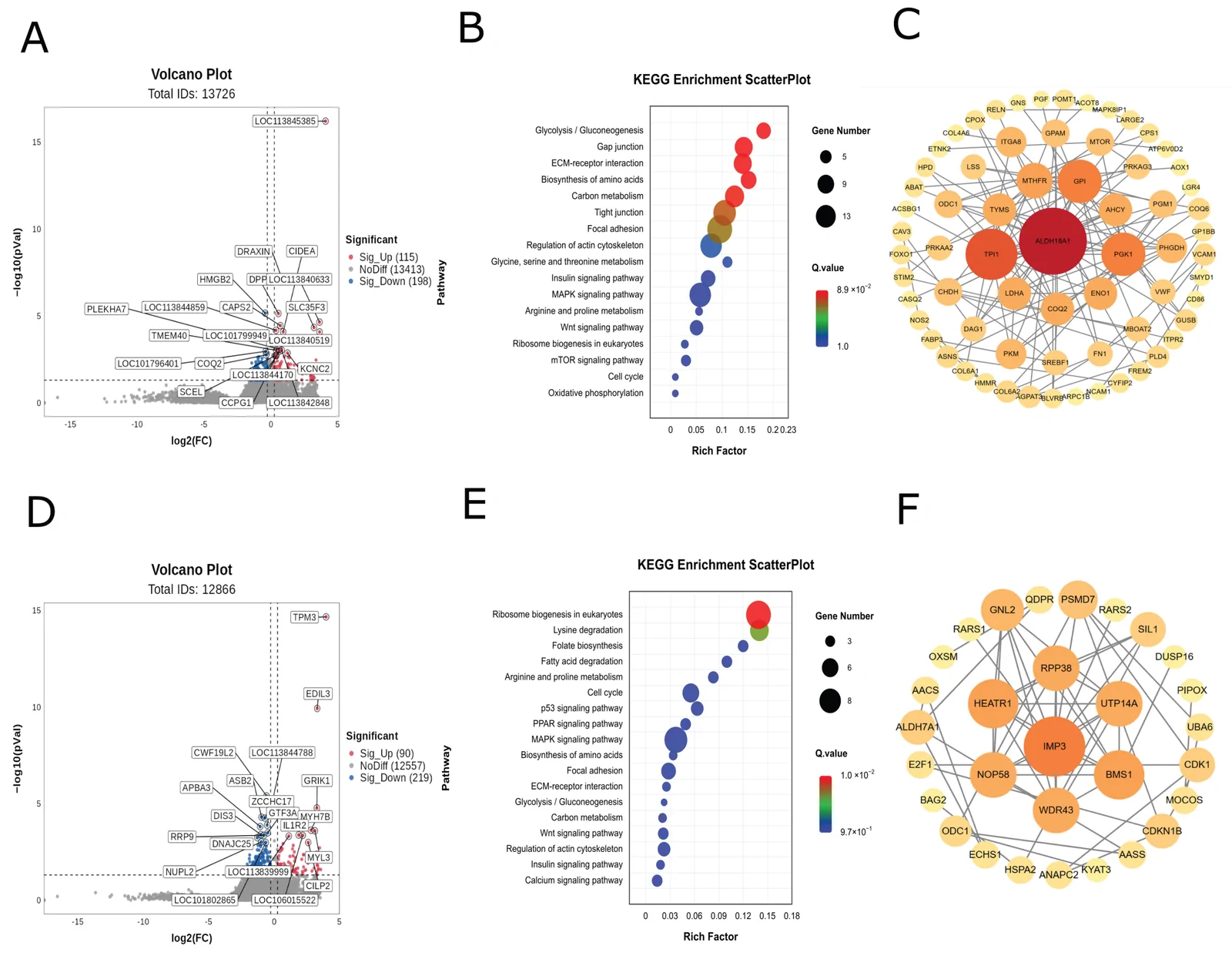
Differential gene expression analysis for high and low breast muscle weight groups. (**A**) Volcano plot of differentially expressed genes (DEGs) in male ducks. (**B**) Bubble plot of KEGG pathway enrichment for DEGs in male ducks. Bubble size represents the number of enriched genes, and the color gradient corresponds to the q-value. (**C**) Protein–protein interaction (PPI) network of DEGs in male ducks. Node size and color depth indicate gene connectivity degree. (**D**) Volcano plot of DEGs in female ducks. (**E**) Bubble plot of KEGG pathway enrichment for DEGs in female ducks. Bubble size represents the number of enriched genes, and the color gradient corresponds to the q-value. (**F**) PPI network of DEGs in female ducks. Node size and color depth indicate gene connectivity degree.

Principal component analysis (PCA) was applied to examine the overall gene expression profile of breast muscle samples from the MH and ML groups (Figure S2A). Hierarchical clustering analysis of differentially expressed genes (DEGs) was performed to visualize expression variation between high and low breast muscle weight subgroups in male ducks, and the male DEG clustering heatmap is displayed in Figure S3A. Differentially expressed genes (DEGs) were screened with a threshold of |log_2_FC| ≥ log_2_ (1.2) and *p* < 0.05 (Figure 4A). A total of 313 DEGs were identified between the MH and ML breast muscle groups, including 115 upregulated genes and 198 downregulated genes. Among the upregulated genes, *CIDEA*, *HMGB2*, *CAPS2*, and *DRAXIN* are closely associated with skeletal muscle myocyte differentiation and muscle satellite cell proliferation. The downregulated genes *COQ2* and *PLEKHA7* exert inhibitory effects on myofiber hypertrophy.

To elucidate the core biological pathways involved in these DEGs, KEGG pathway enrichment analysis was performed (Figure 4B). The results showed that DEGs were significantly enriched in pathways related to energy supply, cytoskeleton, and biosynthesis. Among them, energy and substance metabolism pathways, including glycolysis/gluconeogenesis, carbon metabolism, and biosynthesis of amino acids, exhibited the highest enrichment levels. The mTOR signaling pathway, insulin signaling pathway, MAPK signaling pathway, and Wnt signaling pathway were also enriched. The mTOR and insulin signaling pathways dominate skeletal muscle protein synthesis and myofiber hypertrophy, while the MAPK and Wnt signaling pathways participate in muscle satellite cell proliferation, differentiation, and myocyte development. Meanwhile, pathways regulating myocyte cytoskeleton and cell–cell interaction, such as focal adhesion and ECM-receptor interaction, were also significantly enriched.

To further identify core key genes regulating breast muscle phenotype, a protein–protein interaction (PPI) network was constructed based on proteins encoded by all DEGs (Figure 4C). In the network, nodes represent proteins encoded by DEGs, and edges represent direct interactions between proteins. Node size corresponds to gene connectivity; higher connectivity indicates that the gene acts as a core hub in the regulatory network. Hub genes with high connectivity were screened in this study, including *ALDH9A1*, *GPI*, *PGK1*, *TPI1*, and *ENO1*. These genes are all annotated to energy metabolism pathways such as glycolysis, carbon metabolism, and redox processes, and are also involved in growth regulatory signaling pathways including mTOR, insulin, MAPK, and Wnt, which is consistent with the results of KEGG enrichment analysis. Collectively, the results of pathway enrichment and PPI network indicate that the phenotypic difference in breast muscle weight is synergistically regulated by energy metabolism, protein biosynthesis, and myocyte cytoskeleton development pathways. Core hub genes such as *ALDH9A1*, *GPI*, and *PGK1* are key candidates mediating breast muscle energy supply and myocyte growth and differentiation, providing priority targets for subsequent dissection of the molecular mechanism underlying breast muscle growth and development in male ducks.

As for female samples, PCA was performed to assess the overall gene expression profile of breast muscle samples from the FH and FL groups (Figure S2B). Similarly, hierarchical clustering was carried out on female DEGs, and the corresponding heatmap is presented in Figure S3B. A total of 309 DEGs were identified between the FH and FL breast muscle groups, including 90 upregulated genes and 219 downregulated genes. The DEG clustering heatmap clearly distinguished the two groups, with differentially expressed genes presenting a group-specific expression pattern (Figure 4D).

Among the upregulated genes, *MYH7B* and *TPM3* are key genes governing skeletal muscle structure and myofiber differentiation, and *EDIL3* participates in the regulation of angiogenesis. The significantly downregulated gene *ASB2* mediates muscle protein degradation, and its low expression can reduce muscle protein loss.

KEGG enrichment analysis showed that DEGs were enriched in pathways associated with muscle protein deposition, amino acid homeostasis, and myocyte proliferation (Figure 4E). The most prominent enrichment was observed in pathways including ribosome biogenesis in eukaryotes, lysine degradation, and folate biosynthesis. Pathways such as fatty acid degradation, the MAPK signaling pathway, and the insulin signaling pathway were also involved in regulating breast muscle cell development. Furthermore, a protein–protein interaction (PPI) network was constructed based on the DEGs (Figure 4F). Hub genes with high connectivity were screened, including *IMP3*, *HEATR1*, and *BMS1*, whose core functions are concentrated in ribosome assembly and amino acid metabolism. Collectively, these results indicate that the phenotypic difference in breast muscle between the FH and FL groups is synergistically regulated by protein synthesis, amino acid metabolism, and myocyte proliferation pathways. Hub genes such as *IMP3* can serve as key candidate molecules regulating breast muscle protein deposition in female ducks.

### 3.4. Untargeted Metabolomics Data Analysis

Untargeted metabolomics detection was performed using liquid chromatography-high-resolution mass spectrometry (LC-MS). Equal volumes of all samples were pooled to prepare quality control (QC) samples for monitoring instrumental stability. Metabolite abundance profiles of QC samples were extracted, and a correlation heatmap of QC samples was generated based on Pearson’s correlation coefficient. The results showed that the correlation coefficients among all QC samples in both male and female duck cohorts were higher than 0.97, indicating excellent reproducibility of QC samples. This confirmed stable instrumental performance throughout the mass spectrometry detection process and high data reliability, supporting subsequent differential metabolite screening. A total of 12,600 metabolic ions and 942 MS2-annotated metabolites were identified in the MH and ML groups; for the FH and FL groups, 12,554 metabolic ions and 940 MS2-annotated metabolites were detected.

**Figure 5:**
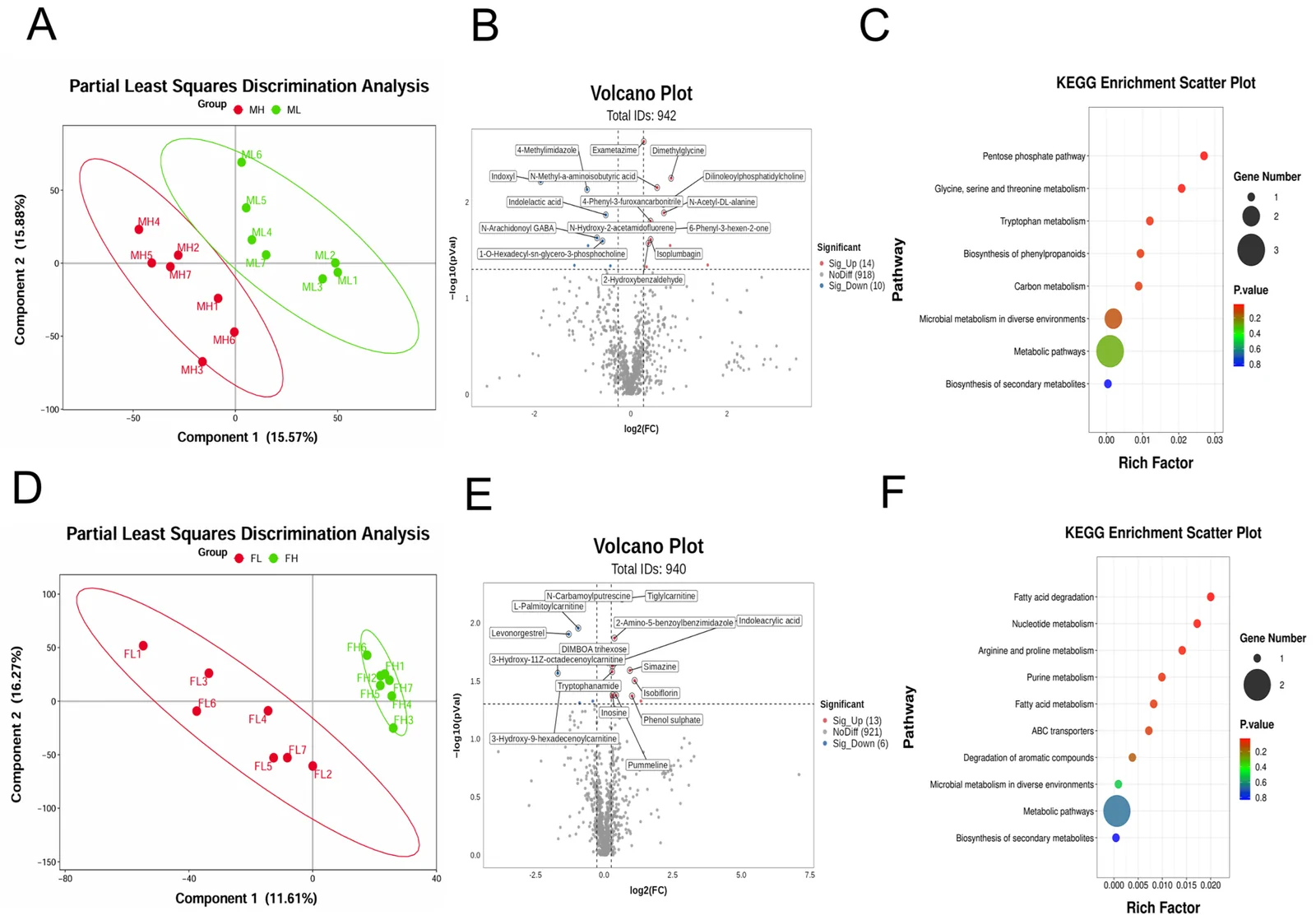
Analysis of differential metabolites between high and low breast muscle weight groups. (**A**) Score plot of PLS-DA for breast muscle metabolomic profiles of male ducks. (**B**) Volcano plot showing differentially abundant metabolites in male ducks. (**C**) Bubble plot of KEGG pathway enrichment for male ducks. (**D**) Score plot of PLS-DA for breast muscle metabolomic profiles of female ducks. (**E**) Volcano plot showing differentially abundant metabolites in female ducks. (**F**) Bubble plot of KEGG pathway enrichment for female ducks. In the volcano plots, red dots represent significantly upregulated metabolites, and blue dots represent significantly downregulated metabolites. In the bubble plots, bubble size corresponds to the number of differential metabolites within each pathway, and deeper red color indicates higher statistical significance of pathway enrichment.

Partial least squares discriminant analysis (PLS-DA) models were constructed for the metabolomics data of the male (MH vs. ML) and female (FH vs. FL) groups, respectively (Figure 5A,D). The PLS-DA score plots revealed tight intra-group clustering of samples with high and low breast muscle weight in both populations, with a distinct inter-group separation trend. Good reproducibility was observed within each group, indicating that metabolic phenotypes can effectively distinguish populations with divergent breast muscle performance.

To avoid model overfitting, permutation tests were performed to validate the reliability of the two PLS-DA models (Figure S4). The intercepts of the Q2 regression lines from permutation tests were both negative, and the Q2 values of the original models were significantly higher than those of randomly permuted models. This verified that the models were stable, valid and free of overfitting, and were suitable for subsequent differential metabolite screening.

A total of 24 differential metabolites (DEMs) were identified in breast muscle between the MH and ML groups, including 14 upregulated and 10 downregulated metabolites (Figure 5B). For the FH and FL comparison, 19 DEMs were detected, with 13 upregulated and six downregulated metabolites (Figure 5E).

Based on the breast muscle DEMs screened by LC-MS metabolomics, KEGG pathway enrichment analysis was conducted for male and female ducks separately. For the MH vs. ML comparison, significantly enriched pathways were mainly involved in carbon metabolism, carbohydrate metabolism, and amino acid anabolism. Pathways including the pentose phosphate pathway, glycine, serine and threonine metabolism, tryptophan metabolism, and carbon metabolism showed higher enrichment factors and stronger enrichment significance. In addition, pathways such as biosynthesis of phenylpropanoids and biosynthesis of secondary metabolites were also enriched (Figure 5C). The pentose phosphate pathway and carbon metabolism act as core pathways for cellular energy supply, while glycine, serine, and threonine provide amino acid precursors for muscle protein synthesis. These results indicate that the phenotypic difference in breast muscle between the MH and ML groups is primarily driven by carbohydrate energy metabolism and basal amino acid turnover. Key intermediates of carbohydrate metabolism, namely 6-phosphogluconic acid, were downregulated; the amino acid metabolism-related metabolite dimethylglycine and the secondary metabolite 2-hydroxybenzaldehyde were upregulated; and the tryptophan metabolism-related metabolite indoxyl was downregulated (Table 3). These molecular changes co-regulate carbohydrate energy metabolism, amino acid homeostasis, and antioxidant processes in the breast muscle of the MH and ML groups.

For the FH vs. FL comparison, DEMs were predominantly enriched in lipid metabolism, arginine/proline muscle anabolism, and nucleotide metabolism. Highly enriched pathways included fatty acid degradation, fatty acid metabolism, arginine and proline metabolism, purine metabolism, and nucleotide metabolism (Figure 5F). Arginine and proline are core amino acids for muscle collagen synthesis and myofiber repair; fatty acid metabolism is involved in intramuscular fat deposition and energy homeostasis; and nucleotide metabolism supports cell proliferation and protein translation. These findings suggest that breast muscle phenotypic divergence in female ducks is mainly associated with metabolic processes related to muscle protein deposition, lipid metabolism, and cell proliferation. The lipid transport molecule L-palmitoylcarnitine was downregulated, while muscle synthesis-related N-carbamoylputrescine and cell proliferation-related inosine were upregulated (Table 3). These molecules jointly modulate lipid metabolism homeostasis, muscle protein synthesis, and cell proliferation in the breast muscle of the FH and FL groups.

Comparison of the enrichment results between the two sexes revealed that DEMs in male breast muscle are centered on carbohydrate and basal amino acid energy metabolism, whereas those in female breast muscle focus on lipid metabolism, muscle-specific amino acids, and cell proliferation-related metabolic processes, indicating prominent sex specificity in the metabolic regulation of breast muscle development.

### 3.5. Multi-Omics Integrated Analysis

**Figure 6:**
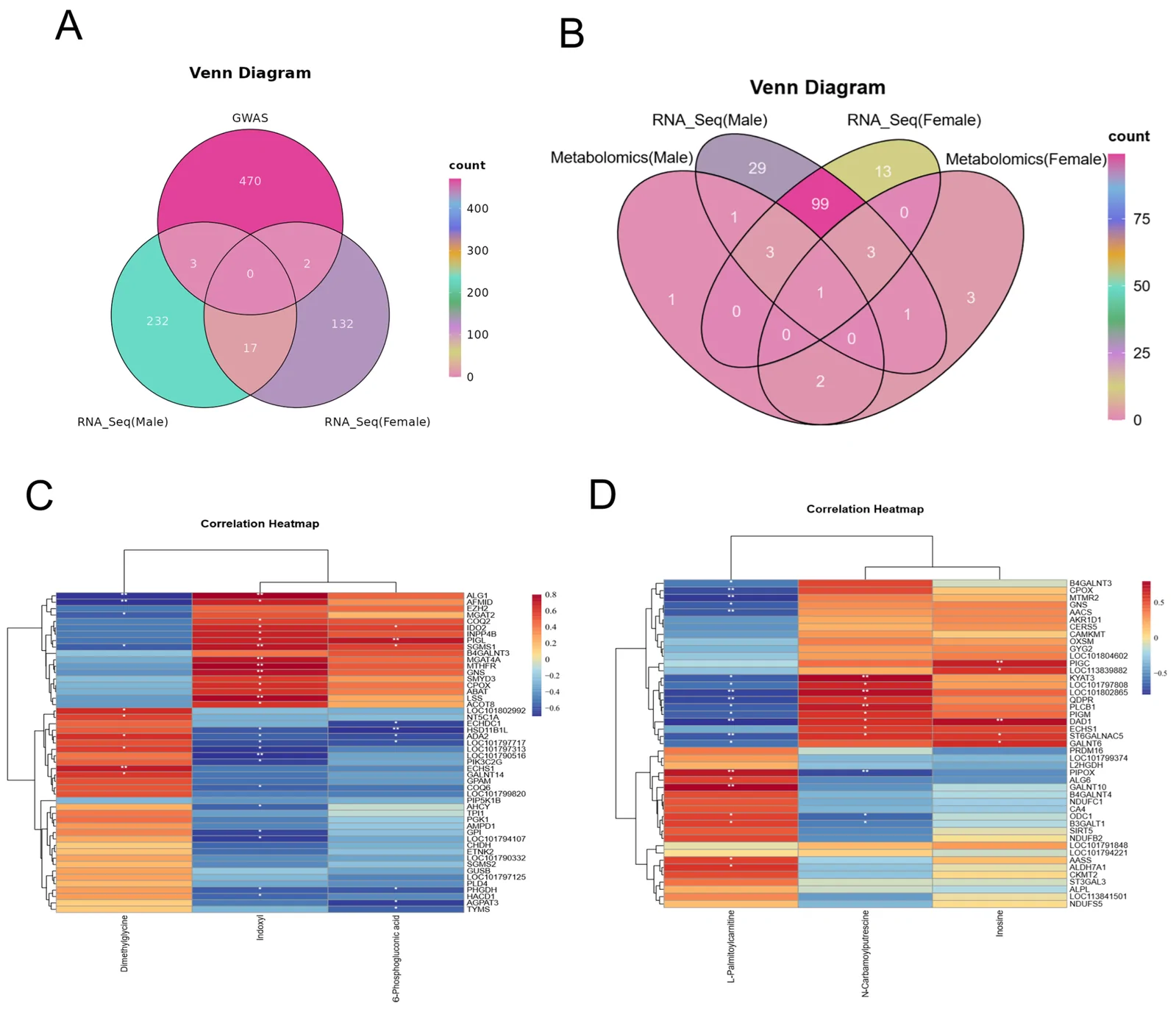
Multi-omics intersection and correlation analyses. (**A**) Venn diagram showing the intersection between GWAS candidate genes and differentially expressed genes (DEGs) from breast muscle. (**B**) Venn diagram of overlapping significantly enriched KEGG pathways derived from transcriptome and metabolome data. (**C**,**D**) Correlation heatmaps between DEGs and differential metabolites. (**C**) Heatmaps showing male ducks with high/low breast muscle weight (MH/ML), and (**D**) female ducks with high/low breast muscle weight (FH/FL). In heatmaps, the *x*-axis represents three core differential metabolites, and the *y*-axis shows key pathway-related DEGs. Red and blue colors indicate positive and negative correlations, respectively. Asterisks mark significantly correlated gene–metabolite pairs, and the side clustering trees reveal hierarchical clustering patterns of molecular correlations.

To narrow down the candidate genes identified by GWAS and pinpoint key functional genes with both genomic variation effects and differential expression in breast muscle, intersection analysis was performed between the full set of significantly associated GWAS candidate genes and the differentially expressed genes (DEGs) of breast muscle in male and female ducks, respectively (Figure 6A). For the male duck group, a total of 252 DEGs were identified from the breast muscle transcriptome, and the non-redundant set of GWAS candidate genes across 10 growth and carcass traits comprised 475 genes. The intersection of the two datasets yielded three shared key genes: *LOC101790109*, *PGP*, and *RAB11FIP3*. For the female duck group, 151 DEGs were screened from the breast muscle transcriptome. After intersection with GWAS candidate genes, two shared key genes were obtained: *SIL1* and *PLCB1*. A total of 17 overlapping DEGs were shared between the male and female DEG sets. Among them, *IL1RW*, *FGF16*, *SESN3*, *DUSP16*, *ECHS1*, *TSHB*, and *CRHR2* are core conserved genes involved in the regulation of skeletal muscle development, energy metabolism, and endocrine signaling. These intersecting genes are core candidates underlying the phenotypic divergence of breast muscle growth traits between male and female Zhongshan Partridge ducks, and can serve as priority targets for further functional validation.

To investigate the correlation between transcriptomic and metabolomic profiles, intersection analysis was conducted on significantly enriched KEGG pathways (*p* < 0.05) derived from differentially expressed genes and MS2-annotated differential metabolites, respectively (Figure 6B). In the male duck group, five shared KEGG pathways were identified, including the pentose phosphate pathway, glycine, serine and threonine metabolism, tryptophan metabolism, carbon metabolism, and metabolic pathways, involving 98 differentially expressed genes and 3 MS2-annotated differential metabolites (Table 4). In the female duck group, four shared KEGG pathways were detected, namely fatty acid degradation, arginine and proline metabolism, fatty acid metabolism, and metabolic pathways, involving 53 differentially expressed genes and three MS2-annotated differential metabolites (Table 4). Metabolic pathways were the only pathway commonly enriched across all four experimental groups, suggesting that basal substance and energy metabolism may act as the shared fundamental mechanism governing breast muscle growth and development in both male and female Zhongshan Partridge ducks.

Spearman correlation analysis was performed between the top 50 genes and the three screened differential metabolites in the male group (Figure 6C). Dimethylglycine showed significantly positive correlations with genes such as *PHGDH*, *CHDH*, *ALG1*, and *MGAT2*. The key amino acid metabolism genes *PHGDH* and *CHDH* are rate-limiting genes for muscle amino acid synthesis, which can promote the synthesis of muscle protein precursors and enhance the protein deposition capacity of breast muscle. Indoxyl was significantly negatively correlated with *AFMID*, *SGM5*, *B4GALTN3*, and *COQ2*. The tryptophan metabolism gene *AFMID* can reduce indoxyl content and alleviate indoxyl-mediated oxidative stress injury in myocytes. 6-Phosphogluconic acid was significantly negatively correlated with *GPI*, *PGM1*, *ETNK2*, and *PYGL*. *GPI* and *PGM1*, core genes in carbohydrate metabolism pathways, can reshape the carbohydrate energy metabolic flux of breast muscle by accelerating the decomposition of carbohydrate intermediates.

Spearman correlation analysis was conducted between the top 50 genes and the three differential metabolites in the female group (Figure 6D). L-Palmitoylcarnitine was significantly negatively correlated with *B4GALNT3*, *CPOX*, *MTMR2*, and *AACS*. *AACS* is a key rate-limiting enzyme for fatty acid synthesis; its high expression inhibits the accumulation of the lipid transporter L-palmitoylcarnitine, reduces intramuscular fatty acid catabolism, and promotes intramuscular lipid deposition. N-Carbamoylputrescine showed significantly positive correlations with *PLCB1*, *QDR*, *PIGM*, and *DAD1*. *PLCB1* is involved in myocyte proliferation and muscle development signaling pathways; its high expression can significantly increase N-carbamoylputrescine content and accelerate myofiber repair and synthesis. *PIGM* regulates the integrity of the myocyte membrane and supports normal myofiber growth. Inosine was positively correlated with *OXSM*, *GYG2*, *CERS5*, and *AKR1D1*. *GYG2* regulates muscle glycogen reserve, and *OXSM* participates in mitochondrial energy metabolism. The two act synergistically with inosine to elevate the energy level of breast muscle cells and promote myocyte proliferation. *CERS5* maintains myocyte homeostasis and reduces oxidative damage.

## 4. Discussion

### 4.1. Phenotypic Characteristics and Growth Patterns of Zhongshan Partridge Ducks

With the aim of systematically assessing the growth-related performance in the unique indigenous breed Zhongshan Partridge ducks, this study is the first to quantitatively analyze body weight and carcass traits across all growth stages, covering five key time points from hatching to 70 days of age, along with carcass weight and breast muscle weight. Growth curve fitting and correlation analysis were applied to characterize the unique growth and developmental patterns of this indigenous duck breed. Currently, most waterfowl studies focus on commercial white-feathered meat ducks, and there is a lack of systematic, comprehensive phenotypic datasets supporting research on growth and carcass traits in Zhongshan Partridge ducks. The phenotypic dataset constructed in this study, combined with subsequent integrated analysis of GWAS, RNA-seq, and metabolomics, reveals the genetic and regulatory mechanisms underlying body weight and breast muscle weight in Zhongshan Partridge ducks, providing unique resources for germplasm conservation and breeding of Zhongshan Partridge ducks. The identified candidate genes, functional pathways and altered metabolic signatures associated with pectoral muscle divergence further supported our scientific hypothesis that divergent molecular regulation governs high and low breast muscle weight phenotypes in this indigenous duck, offering systematic molecular evidence for understanding duck pectoral muscle development.

Consistent with previous multi-omics investigations on indigenous duck breast muscle development, our work identified pathways related to energy metabolism, muscle cell growth, and lipid turnover as central drivers of divergent pectoral muscle performance [26]. Similar multi-omics strategies applied in other native duck populations have demonstrated that combining GWAS, transcriptome and metabolome data is powerful for uncovering candidate genes and metabolites underlying muscle-related traits, which supports the reliability of our analytical framework [27]. Several comparable studies on Chinese local duck breeds also reported that energy-related metabolic shifts represent key molecular signatures distinguishing high and low muscle yield individuals, partially coinciding with the functional observations obtained in the present study [28]. Nevertheless, breed-specific genetic backgrounds lead to distinct candidate gene sets across different indigenous duck resources, highlighting the necessity to carry out population-specific multi-omics dissection for Zhongshan Partridge ducks.

Nonlinear growth models were used to fit the body weight growth trajectory of the studied duck population. The results showed that the Gompertz model exhibited the highest goodness of fit and could accurately reflect the growth characteristics of ducks, that shows rapid growth in the early stage and decelerated growth in the late stage. This finding is consistent with similar studies on indigenous duck breeds such as Pekin ducks, Muscovy ducks, and Loumen ducks [29,30,31], confirming that the Gompertz model is a reliable tool for describing avian growth patterns. Its model parameters can be used to evaluate livestock growth potential and optimize feeding management schemes. Compared to other duck breeds, fast-growing Pekin ducks have a growth inflection point at approximately 3.5 weeks of age, with 35–42 days of age being the optimal marketing period [31,32]. As an indigenous breed, Zhongshan Partridge ducks show a relatively moderate growth rate and gradually enter a growth plateau after 70 days of age. This is consistent with the characteristics of indigenous breeds, with a longer growth cycle and more sufficient meat quality deposition in the late growth stage. Correlation analyses revealed strong positive correlations between BW70 and both CW and BMW, indicating that BW70 can serve as a reliable indirect indicator for slaughter performance. Given that body weight at 70 days can be accurately predicted from early-stage growth records using the fitted growth model, early selection for slaughter and breast muscle traits can be achieved based on early body weight data without waiting for slaughter measurement at 70 days of age. These growth model parameters enable early prediction of ideal slaughter traits, providing a practical reference for optimizing breeding schemes of Zhongshan Partridge ducks.

### 4.2. GWAS Candidate Genes and Population Characteristics

GWAS analyses were performed for growth traits at different stages and breast muscle weight, and three key candidate genes, *FTO*, *ACVR2B*, and *IGFBP3*, were highlighted, corresponding to three core growth dimensions: early development, rapid growth in the middle-late stage, and targeted muscle deposition, reflecting the stage specificity of genetic regulation of growth traits.

We showed that *FTO* is an important gene regulating energy metabolism and embryonic development, and its genetic variants widely affect animal birth weight phenotypes [33]. In this study, *FTO* was mapped to the significantly associated loci for hatch weight, suggesting that it potentially contributes to hatch weight of meat ducks by regulating neonatal developmental processes. Previous studies have shown that *FTO* is highly expressed in muscle tissue during the middle and late stages of embryonic development, with significantly higher expression levels in fast-growing breeds. Its SNPs are significantly associated with chicken hatch weight. At the molecular level, FTO regulates the expression of embryonic development-related genes via m^6^A modification, thereby affecting cell proliferation and differentiation [34]. The results of this study supplement the genetic basis of embryonic body weight regulation in waterfowl.

*ACVR2B* is a transmembrane receptor of the TGF-β superfamily and serves as the core functional receptor for myostatin-mediated negative regulation of muscle growth. In this study, *ACVR2B* was located in the significantly associated interval for AWG-28d to 42d, a critical period for rapid skeletal muscle deposition in meat ducks, and the trait localization is highly consistent with gene function [35]. Functional studies in chickens have shown that silencing *ACVR2B* exerts a stronger enhancing effect on body weight and breast muscle weight than silencing *MSTN*, and its gene polymorphisms are significantly associated with carcass traits [36], making it a key candidate gene for weight gain traits at this stage.

Moreover, *IGFBP3* is a key regulator of the IGF signaling system, which participates in skeletal muscle proliferation, differentiation, and myofiber growth by modulating the bioavailability of IGF-1. In this study, *IGFBP3* was annotated in the candidate interval for breast muscle weight, suggesting it as a functional candidate gene regulating breast muscle weight in meat ducks. Previous studies have confirmed that genetic variants in *IGFBP3* are significantly associated with chicken breast muscle weight and body weight, and its temporal expression coincides with the rapid developmental stage of breast muscle. As for its function, it can regulate myoblast processes through both IGF-dependent and IGF-independent pathways. Given that *IGFBP3* has been shown to exert dual regulatory effects on myoblasts, inhibiting proliferation while promoting differentiation and that its promoter polymorphisms are significantly associated with breast muscle weight in poultry [37], it is speculated that variants in *IGFBP3* can alter the local activity of the IGF pathway in breast muscle, affect myofiber development, and ultimately lead to individual differences in breast muscle weight. Thus, *IGFBP3* can serve as a strong candidate for breeding of breast muscle traits. Notably, although *FTO*, *ACVR2B*, and *IGFBP3* were identified as positional candidate genes from GWAS, they exhibited no significant expression differences between high- and low breast muscle groups in breast muscle RNA-seq (Figure S1). Given the individuals (*n* = 28) with both genotype and expression data, the sample size was insufficient for a robust eQTL analysis. Furthermore, large-scale eQTL resources analogous to the mammalian GTEx project are still under development for duck species. The availability of comprehensive duck multi-tissue eQTL datasets will help resolve the regulatory mechanisms of these positional GWAS candidate genes in future investigations.

Nevertheless, we acknowledge the limited population size (*n* = 365) for GWAS analyses, which is relatively small compared with GWAS studies on growth in commercial meat ducks. This is directly related to the background of the breed. The Zhongshan Partridge ducks used in the experiment are an indigenous protected duck breed that has been rejuvenated over limited generations, making it difficult to obtain a large-scale experimental population. Despite the limited sample size, the current population has a relatively uniform genetic background yet a clear genetic structure, which can effectively reduce the interference of population stratification on association results and ensure the reliability of GWAS mapping.

### 4.3. Sexual Dimorphism in Multi-Omics Regulatory Pathways of Breast Muscle Growth

Sexual dimorphism in breast muscle growth and development is a key phenotypic trait of meat ducks, which involves systematic divergence in metabolic networks and gene regulatory mechanisms. To decipher the molecular basis of this difference, KEGG pathway enrichment analysis was performed on DEGs in breast muscle of male and female ducks respectively, followed by integrated pathway validation combined with differential metabolites.

Functional enrichment revealed remarkable functional differentiation of regulatory pathways between male and female breast muscle. In male ducks, enriched pathways were concentrated in energy and substance metabolism pathways such as glycolysis/gluconeogenesis, carbon metabolism, and biosynthesis of amino acids, suggesting that the rapid growth of male breast muscle relies heavily on energy supply from the glycolytic pathway, and active anabolism provides sufficient precursors for myofiber hypertrophy and rapid muscle protein deposition [38]. Meanwhile, cellular structural pathways including ECM-receptor interaction, focal adhesion, and actin cytoskeleton regulation were significantly enriched, providing a structural basis for myofiber thickening and muscle tissue morphogenesis [39]. Co-enrichment of classic growth signaling pathways such as Wnt, MAPK, insulin, and mTOR indicates that multiple signaling pathways synergistically regulate myoblast proliferation and differentiation, collectively driving the rapid growth of breast muscle in male ducks [40,41].

In contrast to male ducks, the most prominently enriched pathway in female breast muscle was ribosome biogenesis in eukaryotes. Ribosome biogenesis is a core, highly energy-consuming biological process in cells. The efficiency of ribosome biogenesis directly determines the overall translational capacity of cells and further modulates skeletal muscle protein deposition and myofiber hypertrophy [42]. The activity of the ribosome pathway in skeletal muscle exhibits cell-type and breed specificity, and translational regulation is a critical mechanism underlying phenotypic differences in muscle development. These findings suggest that breast muscle growth in female ducks depends more on translational regulation; it enhances muscle protein translation efficiency by boosting ribosome biosynthesis, thereby promoting myofiber hypertrophy. At the metabolic level, differentially expressed genes in female ducks were mainly enriched in pathways such as fatty acid degradation, lysine degradation, and folate biosynthesis, indicating that their energy supply is more dependent on the fatty acid oxidation pathway, and epigenetic regulation mediated by one-carbon metabolism participates in muscle development, which is consistent with the physiological feature of more active intramuscular fat deposition in female ducks [43]. Enrichment of the p53, PPAR, and calcium signaling pathways also reflects that breast muscle development in female ducks focuses more on cell homeostasis maintenance and fine-tuning of lipid metabolism.

Integrated RNA-seq and metabolomic analysis further verified the preferential differences in metabolic pathways between the two sexes. Five commonly significantly enriched pathways were identified in male ducks, mainly involved in carbohydrate and amino acid metabolism; four commonly significantly enriched pathways were found in female ducks, dominated by fatty acid metabolism and amino acid metabolism. In male ducks, key genes of the pentose phosphate pathway, such as *GPI* and *PGM1*, were upregulated, accompanied by altered levels of the intermediate metabolite 6-phosphogluconic acid. This indicates that glucose metabolic flux is shifted toward the pentose phosphate pathway, which generates *NADPH* and ribose-5-phosphate to meet the biosynthetic demands of rapid myocyte proliferation [44]. Activation of the glycine, serine and threonine metabolism pathway and accumulation of dimethylglycine provide methyl donors for protein synthesis via one-carbon metabolism, further supporting muscle protein deposition [45].

In female ducks, differential expression of key enzyme genes in the fatty acid metabolism pathway, including *ECHS1* and *ALDH7A1*, together with changes in L-palmitoylcarnitine content, directly reflects the activity intensity of the fatty acid oxidation pathway. This not only provides sustained energy support for breast muscle growth in female ducks, but also participates in regulating intramuscular fat deposition levels [46]. Upregulation of *ODC1* in the arginine and proline metabolism pathway drives polyamine synthesis, which promotes myoblast proliferation and myofiber hypertrophy; *CKMT2* ensures stable energy supply via the creatine energy shuttle system [47,48].

Cross-group comparison revealed that pathways including the MAPK signaling pathway, Wnt signaling pathway, and focal adhesion were enriched in both male and female ducks, constituting the conserved core regulatory network of duck breast muscle development. However, obvious divergence exists between the two sexes in terms of energy metabolism preference and regulatory hierarchy: male ducks adopt a rapid growth pattern dominated by glycolysis, while female ducks follow a steady growth pattern coordinated by translational regulation and lipid metabolism. This sexual dimorphism in pathway regulation explains the phenotypic divergence in growth rate and muscle quality between male and female ducks at the molecular level.

### 4.4. Candidate Genes Mediating Sex Differences in Breast Muscle Growth

Based on the pathway enrichment and multi-omics correlation analysis, we further identified key regulatory molecules including *ECHS1*, *PGP*, *PLCB1*, and the transcription factor *PRDM16*. Among them, *PGP* and *PLCB1* were supported by evidence from both the GWAS–DEG intersection and gene–metabolite correlation network, while *ECHS1* and *PRDM16* were derived from Spearman correlation analysis between the top 50 genes and differential metabolites.

We demonstrated that *ECHS1* was significantly differentially expressed between the high and low breast muscle groups in both male and female ducks, suggesting a potentially conserved regulatory role in breast muscle growth across sexes. As a key enzyme involved in mitochondrial fatty acid β-oxidation and branched-chain amino acid catabolism [49], *ECHS1* simultaneously modulates the energy production efficiency of both lipid and amino acid metabolic fluxes [50]. During the rapid growth stage of male ducks, it may participate in both amino acid metabolism and carbon metabolism networks, providing sufficient energy and synthetic substrates for myofiber hypertrophy. Therefore, we hypothesized that during the steady growth stage of female ducks, it exerts its energy supply function mainly via the fatty acid degradation pathway. This dual biochemical function provides a potential molecular basis for its sex-specific regulatory pattern.

In male ducks, *PGP* is a critical candidate gene associated with both BW14 and the breast muscle transcriptome, with expression changes synchronized with core glycolytic enzymes such as *GPI* and *PGK1*. Phosphoglycolate phosphatase, encoded by *PGP*, is a key repair enzyme maintaining central carbon metabolism homeostasis. It relieves the inhibitory effects of glycolytic byproducts on the glycolytic and pentose phosphate pathways by degrading these metabolites, thereby ensuring energy supply and biosynthetic efficiency during the rapid proliferation phase of myocytes [51]. Functional studies have confirmed that *PGP* is an essential regulator of skeletal muscle cell proliferation, and its functional deficiency directly leads to cell proliferation arrest and embryonic growth defects [52]. This suggests that differential *PGP* expression may ultimately affect individual body weight and breast muscle weight by modulating glucose metabolic flux in breast muscle cells.

In female ducks, *PLCB1* and the differential metabolite N-carbamoylputrescine were co-enriched in the core regulatory network. Phospholipase C β1, encoded by *PLCB1*, is a key effector of the phosphatidylinositol signaling pathway, which regulates myoblast differentiation by activating intracellular calcium signaling and the β-catenin pathway [53,54]. As an intermediate of arginine metabolism, N-carbamoylputrescine serves as an important precursor for polyamine synthesis and provides a material basis for myocyte proliferation. The synergistic effect of the two indicates that a potential metabolic reprogramming mediated by phospholipid signaling is a vital regulatory mechanism underlying breast muscle growth rate differences in female ducks, forming remarkable sexual dimorphism compared with the male regulatory pattern centered on glucose metabolism.

In addition, the transcription factor *PRDM16*, obtained from gene–metabolite correlation analysis in this study, bridges the regulatory networks of breast muscle growth, metabolic regulation, and sexual dimorphism. PR domain-containing protein 16, encoded by this gene, can act as a core molecular switch governing myogenic precursor cell fate and myofiber type in vertebrates. Its high expression suggests it may activate the fatty acid degradation pathway and promote intramuscular fat deposition, and may potentially inhibit *MyoD*-mediated myofiber hypertrophy [55]. Previous genomic studies have confirmed that *PRDM16* is a key candidate gene regulating skeletal muscle development and organismal growth, and genetic variations in *PRDM16* under different genetic backgrounds can lead to significant divergence in growth and fat deposition [56]. In this study, *PRDM16* was enriched in core metabolic pathways of female duck breast muscle and showed significant expression differences between the high and low breast muscle groups. High *PRDM16* expression found specifically in female ducks is expected to drive the conversion of myofibers to an oxidative phenotype, thus potentially upregulating key fatty acid oxidation enzymes such as *ECHS1*, supporting the steady growth rhythm with sustained energy supply, and thereby increasing intramuscular fat deposition. In contrast, low *PRDM16* expression in male ducks is hypothesized to favor rapid myofiber hypertrophy, matching the fast-growth characteristics of male ducks. The differential regulation of this gene may reflect the sexual dimorphism of breast muscle metabolism.

It should be noted that the functional interpretations for many candidate genes, such as *PLCB1* and *PRDM16*, were largely extrapolated from published results of other species. Although these mechanisms are reported to be relatively conserved in muscle biology, their concrete molecular functions remain to be verified in duck breast muscle. In addition, further metabolic quantification and large-scale validation are needed to verify the key metabolic biomarkers identified. Moreover, further investigation on sex-by-phenotype interaction effects will also help comprehensively uncover the complex molecular regulatory network underlying divergent breast muscle performance in indigenous ducks.

## 5. Conclusions

In this study, we investigated the phenotypic differences and molecular regulatory signals underlying pectoral muscle development, which is critical for the conservation and genetic improvement of indigenous duck genetic resources. We integrated GWAS, RNA-seq and metabolomics to systematically explore growth and pectoral muscle regulatory mechanisms in Zhongshan Partridge ducks, a protected indigenous waterfowl breed. Gompertz model was optimal for fitting its growth trajectory, and BW70 could be used for early slaughter trait selection. Three stage-specific functional genes (*FTO*, *ACVR2B*, *IGFBP3*) were identified via GWAS for growth-related traits. Multi-omics results revealed obvious sex-dimorphic metabolic patterns in breast muscle, with *ECHS1*, *PGP*, *PLCB1* and *PRDM16* acting as potential regulators of gender differences. This study supplements the molecular basis of native duck growth research and provides a foundation for germplasm conservation and breeding of Zhongshan Partridge ducks.

## Figures and Tables

**Table 1:** Comparison of growth and carcass traits between male and female Zhongshan Partridge ducks. All values are presented as mean ± standard deviation in grams (g). Male and Female refer to male and female ducks, respectively. N.S. indicates no significant difference between sexes. Asterisk * denotes *p* < 0.05, and double asterisks ** denote *p* < 0.01. BW1, BW14, BW28, BW42 and BW70 represent body weight at 1, 14, 28, 42 and 70 days of age; AWG means absolute weight gain in different stages; BMW stands for breast muscle weight; CW represents carcass weight.

Trait	Male	Female	Statistical Test
BW1 (g)	47.05 ± 4.31	47.82 ± 4.31	N.S.
BW14 (g)	334.53 ± 53.39	347.86 ± 55.22	*
BW28 (g)	956.07 ± 145.16	973.66 ± 157.21	N.S.
BW42 (g)	1615.08 ± 220.14	1616.30 ± 212.37	N.S.
BW70 (g)	2322.00 ± 261.26	2214.54 ± 205.91	**
AWG-28d (g)	912.23 ± 142.10	928.87 ± 156.04	N.S.
AWG-28d to 42d (g)	654.72 ± 144.67	640.19 ± 148.49	**
AWG-42d to 70d (g)	708.00 ± 182.86	595.86 ± 135.96	**
BMW (g)	232.64 ± 29.60	215.55 ± 25.30	**

**Table 2:** Parameters of three nonlinear growth models (Logistic, Gompertz, von Bertalanffy) for male and female Zhongshan Partridge ducks. The asymptotic body weight and inflection day are expressed as mean ± standard error. Adjusted R2 indicates the goodness-of-fit of each model, and reduced chi-square values are used to comprehensively evaluate the complexity and stability of the models.

Model	Gender	Maximum Body Weight (g)	Inflection Point Age (Days)	Adjusted R^2^	Reduced Chi-Square
Logistic	Male	3125.50 ± 195.88	42.02 ± 3.00	0.99926	0.03601
	Female	2822.71 ± 184.55	38.06 ± 3.07	0.99894	0.06075
Gompertz	Male	2691.81 ± 73.61	28.94 ± 0.74	0.99931	0.03358
	Female	2503.71 ± 69.80	27.07 ± 0.80	0.99908	0.05248
von Bertalanffy	Male	3568.45 ± 647.85	31.81	0.98861	0.55253
	Female	3128.98 ± 472.69	28.29	0.98925	0.61416

**Table 3:** KEGG pathways enriched by differential metabolites and their expression trends in male and female ducks. Columns include gender (Gender), KEGG pathway name (KEGG Path), and differential expression trend (Differential Express Trend). The label up indicates overall significant upregulation of differential metabolites in the pathway, while down indicates overall significant downregulation.

Gender	KEGG Pathway Name	Differential Metabolite Name	Express Trend
Male	Metabolic pathways	Dimethylglycine	up
		6-Phosphogluconic acid	down
		2-Hydroxybenzaldehyde	up
	Glycine, serine and threonine metabolism	Dimethylglycine	up
	Carbon metabolism	6-Phosphogluconic acid	down
	Pentose phosphate pathway	6-Phosphogluconic acid	down
	Tryptophan metabolism	Indoxyl	down
	Biosynthesis of secondary metabolites	6-Phosphogluconic acid	down
	Microbial metabolism in diverse environments	6-Phosphogluconic acid	down
		2-Hydroxybenzaldehyde	up
	Biosynthesis of phenylpropanoids	2-Hydroxybenzaldehyde	up
Female	Metabolic pathways	Inosine	up
		N-Carbamoylputrescine	up
	Arginine and proline metabolism	N-Carbamoylputrescine	up
	Fatty acid metabolism	L-Palmitoylcarnitine	down
	Fatty acid degradation	L-Palmitoylcarnitine	down
	Nucleotide metabolism	Inosine	up
	Purine metabolism	Inosine	up
	ABC transporters	Inosine	up
	Biosynthesis of secondary metabolites	N-Carbamoylputrescine	up
	Microbial metabolism in diverse environments	Fluorobenzene	down
	Degradation of aromatic compounds	Fluorobenzene	down

**Table 4:** Information on differentially expressed genes (DEGs) and differential metabolites (DEMs) corresponding to shared KEGG pathways enriched in breast muscle transcriptome and metabolome datasets.

Gender	KEGG Pathway Name	DEGs	DEMs
Male	Pentose phosphate pathway	*GPI*, *PGM1*	6-Phosphogluconic acid
	Glycine, serine and threonine metabolism	*CHDH*, *LOC101797717*, *PHGDH*, *LOC101802992*	Dimethylglycine
	Tryptophan metabolism	*ECHS1*, *AFMID*, *IDO2*, *AOX1*	Indoxyl
	Carbon metabolism	*ECHS1*, *CPS1*, *PGK1*, *PGP*, *ENO1*, *PHGDH*, *LOC101794107*, *LOC101802992*, *PKM*, *GPI*, *TPI1*	6-Phosphogluconic acid
	Metabolic pathways	*LOC101797313*, *PIK3C2G*, *ADA2*, *GBGT1*, *CHDH*, *COQ2*, *ACSBG1*, *AHCY*, *LOC101797717*, *CPOX*, *ABAT*, *PTS*, *GK*, *SGMS1*, *INPP4B*, *COQ6*, *LOC101790332*, *GALNT14*, *CPS1*, *SMYD3*, *GUSB*, *PIP5K1B*, *HACD1*, *HPD*, *PGK1*, *MBOAT2*, *SGMS2*, *ALG1*, *ACOT8*, *PGP*, *MTHFR*, *PDE7A*, *ENO1*, *LARGE2*, *POMT1*, *ST8SIA5*, *GPAM*, *ASNS*, *AFMID*, *NOS2*, *MGAT2*, *ATP6V0D2*, *LOC101799820*, *B3GAT1*, *PHGDH*, *EZH2*, *AMPD1*, *HSD11B1L*, *LOC101794107*, *LOC101802992*, *PKM*, *IDO2*, *PLD4*, *LOC101797125*, *GNS*, *LOC101792573*, *LOC101790516*, *BLVRB*, *ECHDC1*, *ETNK2*, *GPI*, *B4GALNT3*, *ODC1*, *SMYD1*, *TPI1*, *AGPAT3*, *AOX1*, *ST6GALNAC3*, *TYMS*, *LDHA*, *PGM1*, *LSS*, *PIGL*, *MGAT4A*	Dimethylglycine
Female	Fatty acid degradation	*ECHS1*, *ALDH7A1*, *LOC101791848*	L-Palmitoylcarnitine
	Arginine and proline metabolism	*ODC1*, *CKMT2*, *ALDH7A1*	N-Carbamoylputrescine
	Fatty acid metabolism	*ECHS1*, *OXSM*	L-Palmitoylcarnitine
	Metabolic pathways	*SIRT5*, *PIPOX*, *LOC101802865*, *ECHS1*, *B3GALT1*, *AKR1D1*, *NDUFC1*, *LOC101794221*, *ODC1*, *ALPL*, *PRDM16*, *B4GALNT4*, *AASS*, *CA4*, *CKMT2*, *ALDH7A1*, *LOC113841501*, *ALG6*, *NDUFB2*, *ST3GAL3*, *LOC101799374*, *GALNT10*, *L2HGDH*, *NDUFS5*, *GNS*, *QDPR*, *CERS5*, *PLCB1*, *DAD1*, *MTMR2*, *KYAT3*, *PIGM*, *CPOX*, *AACS*, *GYG2*, *B4GALNT3*, *LOC101791848*, *CAMKMT*, *LOC101797808*, *PIGC*, *OXSM*, *LOC101804602*, *ST6GALNAC5*, *LOC113839882*, *GALNT6*	Inosine

## Data Availability

The original contributions presented in this study are included in the article/Supplementary Materials. Further inquiries can be directed to the corresponding authors. Specifically, partial summarized phenotypic, GWAS, transcriptomic and metabolomic datasets supporting the conclusions of this research are available in the manuscript and Supplementary Materials. Complete raw sequencing files and full original phenotypic records are archived in our laboratory, which can be provided by the corresponding author upon reasonable request.

## Supplementary Materials

The following supporting information can be downloaded at https://www.mdpi.com/article/10.3390/biom16091335/s1. Supplementary Figure S1 Normalized gene expression levels of three GWAS candidate genes (FTO, ACVR2B, and IGFBP3) across four phenotypic groups. The MH represents male high-breast-muscle-weight group, ML represents male low-breast-muscle-weight group, FH represents female high-breast-muscle-weight group, and FL represents female low-breast-muscle-weight group. ns indicates no significant difference between compared groups. Supplementary Figure S2 Principal component analysis (PCA) score plots of metabolomic profiles for male and female ducks with divergent breast muscle weight. (A) Male duck groups (MH/ML); (B) Female duck groups (FH/FL). Ellipses represent the 95% confidence interval, and scatter points with distinct colors denote samples from different groups. The plots were used to evaluate inter-group separation and sample reproducibility within each group. Supplementary Figure S3 Clustering heatmaps of differentially expressed genes (DEGs) between high and low breast muscle weight groups. (A) DEG heatmap for male duck MH/ML groups; (B) DEG heatmap for female duck FH/FL groups. Each row corresponds to a single DEG, and each column represents an individual sample. Color gradient indicates the relative gene expression level, and the dendrogram on the left shows hierarchical clustering of gene expression patterns. Supplementary Figure S4 Permutation test validation plots for metabolomic PLS-DA discriminant models. (A) Permutation test result of the PLS-DA model for male duck high/low breast muscle groups; (B) Permutation test result of the PLS-DA model for female duck high/low breast muscle groups. The x-axis displays the R^2^ and Q^2^ values of permuted models. The blue and red fitted curves correspond to Q^2^ and R^2^, respectively, to assess overfitting of the PLS-DA model. Supplementary Table S1. Nutritional standard specifications of starter duck feed (548) and grower duck feed (545). The table presents core nutritional indices including crude protein, crude fiber, crude ash, total phosphorus and lysine. Values represent the minimum or maximum limit of each index, and all units are mass percentage (%). Supplementary Table S2. Information of significant SNP loci identified by genome-wide association study for ten growth and carcass traits. Columns display trait name (Trait), chromosome number (CHROM), physical position of SNP (POS), allelic variants (Allele), annotated candidate gene (GENE), and association *p*-value. NA indicates no annotated candidate gene for the corresponding SNP. Supplementary Table S3. Phenotypic statistics of different genotypes at significant SNP loci for ten growth and carcass traits.Columns include trait name (Trait), chromosomal physical position of SNP (SNP), genotype (Allele), individual count of each genotype (Count), phenotypic values expressed as mean ± standard error (Weight), and association *p*-values. Different uppercase superscript letters within the same column indicate extremely significant differences (*p* < 0.01), while lowercase superscript letters denote significant differences (*p* < 0.05) between genotypes. Supplementary Table S4. Information of candidate genes annotated from significant SNPs of GWAS for ten growth and carcass traits. Columns include trait name (Trait), gene symbol (Gene), chromosome number (Chr), gene start position (Start), gene end position (End), number of exons (Exon num), number of introns (Intron num), gene strand orientation (Strand), and total number of significant SNPs within each gene (Number of SNP). Supplementary Table S5. Quality control statistics of sequencing data for 28 duck breast muscle metabolome samples. Columns are sample ID (Sample), raw sequencing reads (Raw Data), valid sequencing reads (Valid Data), Q20 base percentage (I Q20%), Q30 base percentage (Q30%), and GC content percentage (GC content%). FH and FL denote female ducks with high and low breast muscle weight, respectively; MH and ML denote male ducks with high and low breast muscle weight, respectively.

## Abbreviations

The following abbreviations are used in this manuscript:

AWG	Average Weight Gain
BW	Body Weight
BMW	Breast Muscle Weight
CW	Carcass Weight
DEGs	Differentially Expressed Genes
DEMs	Differentially Expressed Metabolites
FarmCPU	Fixed And Random Model Circuitous Probability Unification
GWAS	Genome-Wide Association Study
KEGG	Kyoto Encyclopedia of Genes and Genomes
QQ	Quantile–quantile
RNA-seq	RNA Sequencing
SNP	Single Nucleotide Polymorphism
