# Supplementary material for: Integrated Multi-Omic Analyses Reveal Gender-Specific Molecular Mechanisms Regulating Growth and Muscle Development in Zhongshan Partridge Ducks
Source: Biomolecules. 2026 Sep 14;16(9):1335. doi: 10.3390/biom16091335 (PMC13604374; doi:10.3390/biom16091335)
Supplement: Supplementary file 1 [file biomolecules-16-01335-s001.zip › Supplementary_1_All_Figures.pdf]

Supplementary figures

Integrated Multi-Omic Analyses Reveal Gender-Specific Molecular Mechanisms Regulating Growth and Muscle Development in Zhongshan Partridge Ducks

***Corresponding**: Qingbin Luo (qbluo@scau.edu.cn) & Zhou Wu (zhou.wu@scau.edu.cn)

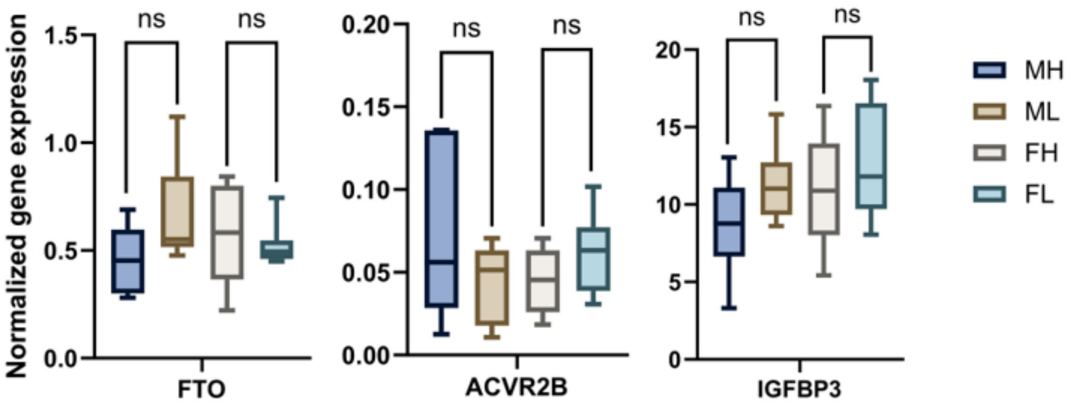

Figure S1. Normalized gene expression levels of three GWAS candidate genes (*FTO*, *ACVR2B*, and *IGFBP3*) across four phenotypic groups. The MH represents male high-breast-muscle-weight group, ML represents male low-breast-muscle-weight group, FH represents female high-breast-muscle-weight group, and FL represents female low-breast-muscle-weight group. ns indicates no significant difference between compared groups.

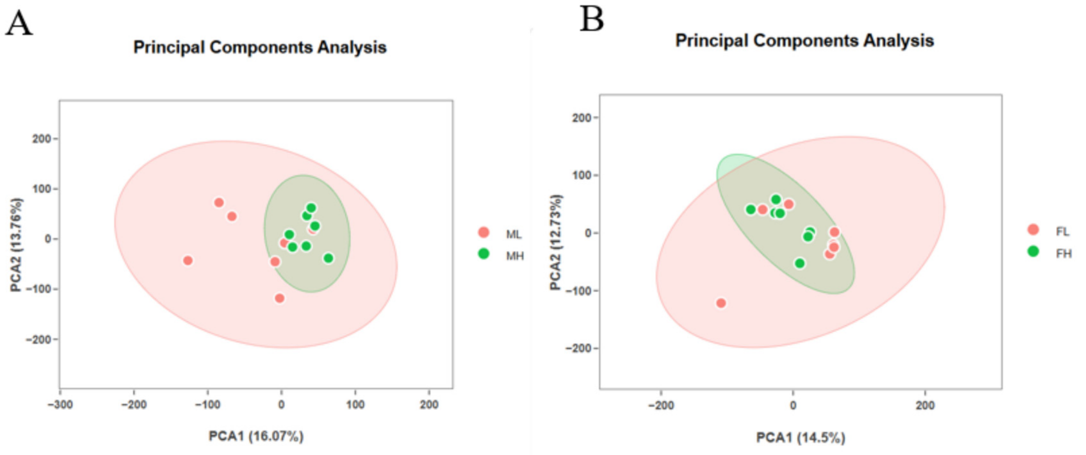

Figure S2. Principal component analysis (PCA) score plots of metabolomic profiles for male and female ducks with divergent breast muscle weight. (A) Male duck groups (MH/ML); (B) Female duck groups (FH/FL). Ellipses represent the 95% confidence interval, and scatter points with distinct colors denote samples from different groups. The plots were used to evaluate inter-group separation and sample reproducibility within each group.

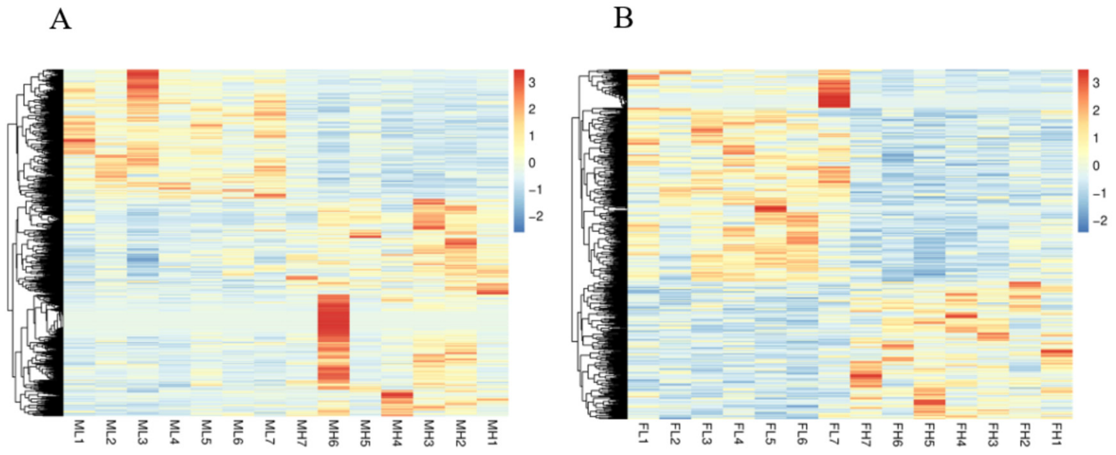

Figure S3. Clustering heatmaps of differentially expressed genes (DEGs) between high and low breast muscle weight groups. (A) DEG heatmap for male duck MH/ML groups; (B) DEG heatmap for female duck FH/FL groups. Each row corresponds to a single DEG, and each column represents an individual sample. Color gradient indicates the relative gene expression level, and the dendrogram on the left shows hierarchical clustering of gene expression patterns.

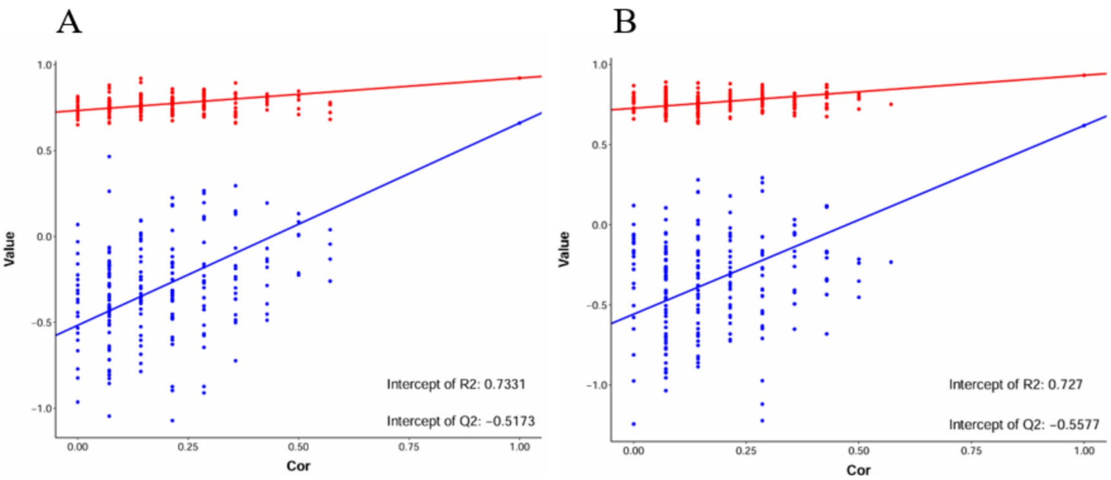

Figure S4. Permutation test validation plots for metabolomic PLS-DA discriminant models. (A) Permutation test result of the PLS-DA model for male duck high/low breast muscle groups; (B) Permutation test result of the PLS-DA model for female duck high/low breast muscle groups. The x-axis displays the R^2 and Q^2 values of permuted models. The blue and red fitted curves correspond to Q^2 and R^2, respectively, to assess overfitting of the PLS-DA model.
